# Changes in Surgical Opioid Prescribing and Patient-Reported Outcomes After Implementation of an Insurer Opioid Prescribing Limit

**DOI:** 10.1001/jamahealthforum.2023.3541

**Published:** 2023-10-13

**Authors:** Kao-Ping Chua, Thuy D. Nguyen, Chad M. Brummett, Amy S. Bohnert, Vidhya Gunaseelan, Michael J. Englesbe, Jennifer F. Waljee

**Affiliations:** 1 Susan B. Meister Child Health Evaluation and Research Center, Department of Pediatrics, University of Michigan Medical School, Ann Arbor; 2 Department of Health Management and Policy, University of Michigan School of Public Health, Ann Arbor; 3 Department of Anesthesiology, University of Michigan Medical School, Ann Arbor; 4 Michigan Opioid Prescribing Engagement Network, University of Michigan Medical School, Ann Arbor; 5 Department of Surgery, University of Michigan Medical School, Ann Arbor

## Abstract

**Importance:**

Insurers are increasingly limiting the duration of opioid prescriptions for acute pain. Among patients undergoing surgery, it is unclear whether implementation of these limits is associated with changes in opioid prescribing and patient-reported outcomes, such as pain.

**Objective:**

To assess changes in surgical opioid prescribing and patient-reported outcomes after implementation of an opioid prescribing limit by a large commercial insurer in Michigan.

**Design, Setting, and Participants:**

This was a cross-sectional study with an interrupted time series analysis. Data analyses were conducted from October 1, 2022, to February 28, 2023. The primary data source was the Michigan Surgical Quality Collaborative, a statewide registry containing data on opioid prescribing and patient-reported outcomes from adults undergoing common general surgical procedures. This registry is linked to Michigan’s prescription drug monitoring program database, allowing observation of opioid dispensing. The study included 6045 adults who were covered by the commercial insurer and underwent surgery from January 1, 2017, to October 31, 2019.

**Exposure:**

Policy limiting opioid prescriptions to a 5-day supply in February 2018.

**Main Outcomes and Measures:**

Among all patients, segmented regression models were used to assess for level or slope changes during February 2018 in 3 patient-reported outcomes: pain in the week after surgery (assessed on a scale of 1-4: 1 = none, 2 = minimal, 3 = moderate, and 4 = severe), satisfaction with surgical experience (scale of 0-10, with 10 being the highest satisfaction), and amount of regret regarding undergoing surgery (scale of 1-5, with 1 being the highest level of regret). Among patients with a discharge opioid prescription and a dispensed opioid prescription (prescription filled within 3 days of discharge), additional outcomes included total morphine milligram equivalents in these prescriptions, a standardized measure of opioid volume.

**Results:**

Among the 6045 patients included in the study, mean (SD) age was 48.7 (12.6) years and 3595 (59.5%) were female. Limit implementation was not associated with changes in patient-reported satisfaction or regret and was associated with only a slight level decrease in patient-reported pain score (−0.15 [95% CI, −0.26 to −0.03]). Among 4396 patients (72.7%) with a discharge and dispensed opioid prescription, limit implementation was associated with a −22.3 (95% CI, −32.8 to −11.9) and −26.1 (95% CI, −40.9 to −11.3) level decrease in monthly mean total morphine milligram equivalents of discharge and dispensed opioid prescriptions, respectively. These decreases corresponded approximately to 3 to 3.5 pills containing 5 mg of oxycodone.

**Conclusions:**

This cross-sectional analysis of data from adults undergoing general surgical procedures found that implementation of an insurer’s limit was associated with modest reductions in opioid prescribing but not with worsened patient-reported outcomes. Whether these findings generalize to other procedures warrants further study.

## Key Points

**Question:**

Is implementation of a commercial insurer’s 5-day opioid prescribing limit associated with a change in patient-reported outcomes, such as pain control, among adult patients undergoing surgery?

**Findings:**

In this cross-sectional analysis of a statewide surgical registry linked to the Michigan prescription drug monitoring program database, limit implementation was associated with modest reductions in opioid prescribing and dispensing but not with worsened patient-reported outcomes among 6045 adults undergoing common general surgical procedures.

**Meaning:**

Findings suggest that the insurer’s limit was not associated with worsened patient-reported outcomes after surgery, but additional studies are needed to determine whether findings generalize to other opioid prescribing limits and surgical procedures.

## Teaser

This cross-sectional study determines whether patient-reported outcomes, such as pain control, changed after implementation of a commercial insurer’s 5-day opioid prescribing limit.

## Introduction

In 2019, US surgeons accounted for 15.5 million opioid prescriptions, constituting 11% of opioid prescriptions dispensed that year.^1^ The true number of opioid prescriptions associated with surgery is even higher because advanced practice clinicians account for one-fifth of opioid prescriptions written after surgery and one-quarter of refills.^2^ Prescription opioid exposure after surgery is associated with an increased risk of opioid-related adverse events, including opioid overdose, misuse, and persistent opioid use, a pattern of continued opioid use after acute surgical pain typically resolves.^3,4,5^ Owing to the prevalence of surgical opioid prescribing and its associated harms, reducing unnecessary surgical opioid prescribing is an important step to mitigate the role of prescription opioids in the US opioid epidemic.

In part to achieve this goal, at least 39 states have implemented policies limiting the duration of opioid prescriptions for acute pain, opioid prescriptions written to patients without prior opioid use, or both.^6,7,8,9^ Many major insurers, such as UnitedHealthcare, Medicare, and several state Medicaid programs, have also implemented opioid prescribing limits.^10,11,12^ To date, several studies have evaluated the association between implementation of state limits and opioid prescribing to patients undergoing surgery, with most finding modest or no reductions in this prescribing.^13,14,15^

Two important knowledge gaps remain. First, few studies have evaluated the association between insurer limits and opioid prescribing after surgery. This is an important gap because insurer and state limits work through different mechanisms. For example, state limits rely on changes in behavior by prescribers and pharmacists. In contrast, insurer limits rely not just on these changes but also on denial of coverage at the point of dispensing, which could result in better enforcement and greater reductions in opioid prescribing, all other factors being equal.

Second, few studies have rigorously evaluated the association between implementation of opioid prescribing limits and patient-reported outcomes after surgery, such as pain.^12^ As noted in a federal report summarizing the literature on opioid prescribing limits,^12^ such studies would require prospective collection of data on patient-reported outcomes before and after limit implementation. However, few groups were collecting such data during the latter half of the 2010s, when most limits took effect.

In this study, we evaluated whether implementation of a 5-day opioid prescribing limit by Blue Cross Blue Shield of Michigan (BCBSM), an insurer that covers approximately three-quarters of privately insured patients in Michigan, was associated with changes in opioid prescribing, opioid dispensing, and patient-reported outcomes among adults undergoing general surgical procedures across the state. To conduct this analysis, we used our access to a statewide surgical registry with data on opioid prescribing and patient-reported outcomes since January 2017, before implementation of the BCBSM limit in February 2018. This surgical registry database is linked to Michigan’s prescription drug monitoring program (PDMP) database, allowing observation of opioid prescribing (via the registry) and opioid dispensing (via the PDMP).

## Methods

### Data Sources

The institutional review board of the University of Michigan Medical School approved this cross-sectional study with a waiver of informed consent because data were deidentified. This study followed the Strengthening the Reporting of Observational Studies in Epidemiology (STROBE) reporting guideline for cross-sectional studies.

Our primary data source was the Michigan Surgical Quality Collaborative (MSQC), an all-payer, statewide surgical registry containing data from adults undergoing general surgical procedures at 70 acute care hospitals across Michigan (see eAppendix 1 in Supplement 1 for included procedures).^16,17^ Key data elements include patient characteristics (eg, age, sex, and payer name), procedure characteristics (eg, procedure type), and whether patients had a discharge opioid prescription, as recorded via medical record review by a trained nurse abstractor. The database does not report days supplied in discharge opioid prescriptions but does report opioid name, strength, and quantity. These data elements, along with published conversion factors, allowed calculation of total morphine milligram equivalents (MMEs), a standardized measure of opioid prescription volume.^18^ Finally, the database reports several patient-reported outcomes, including pain in the week after surgery, satisfaction with surgical experience, and amount of regret regarding undergoing surgery. These data derive from patient surveys sent 30 to 90 days after discharge (see eAppendix 2 in Supplement 1 for details).

Our second data source was the Michigan Automated Prescription System, the state’s PDMP database. This database reports information on every controlled substance prescription dispensed in Michigan pharmacies regardless of method of payment, including cash. Unlike MSQC data, the PDMP includes information on days supplied. The PDMP data are linked to MSQC data by an independent third party using a state-approved process in which patient identifiers are replaced with encrypted identifiers. We used this linkage to identify dispensed opioid prescriptions, defined as opioid prescriptions dispensed within 3 days of discharge from surgery (or within 3 days of discharge from hospitalization after surgery, as applicable).

### Sample

The study period was January 1, 2017, through October 31, 2019 (see eAppendix 3 in Supplement 1 for rationale). We initially included MSQC patients covered by BCBSM who were admitted and discharged from surgery during the study period and had complete data on the occurrence and dosing of discharge opioid prescriptions. If patients had multiple procedures during the study period, we included only the earliest one. We excluded patients who were younger than 18 years, were hospitalized for more than 14 days after surgery or had invalid data on length of stay, were not discharged home after hospitalization (if applicable), died within 30 days of surgery, had a reoperation or readmission within 30 days of surgery, lived out of state, or had multiple matches in the PDMP database. To account for potential data entry error, we excluded patients who had discharge or dispensed opioid prescriptions with total MMEs in the top 1%. To mitigate bias from compositional changes in the sample, we excluded patients from MSQC hospitals that did not collect data on opioid prescribing and patient-reported outcomes during the preintervention period. Finally, we excluded patients without complete data on the 3 patient-reported outcomes assessed in this study.

The final sample included patients undergoing surgery at 1 of 39 hospitals. Early in the study period, some hospitals collected data on opioid prescribing and patient-reported outcomes from only a subset of patients (eAppendix 2 in Supplement 1). Over time, the share of patients surveyed increased. Consequently, the number of patients in the sample was higher in 2018 and 2019 compared with 2017.

### Exposure

On February 1, 2018, BCBSM implemented a policy that limited the duration of new prescriptions for short-acting opioids to a 5-day supply.^19^ BCBSM defined new prescriptions as those that were for patients lacking opioid dispensing in the prior 120 days (hereafter referred to as *opioid-naive patients*).^20^

### Outcomes

We created 2 sets of outcomes. Each set had 5 outcomes. The first set was calculated for all patients and included the occurrence of a discharge opioid prescription (based on MSQC data), the occurrence of a dispensed opioid prescription (based on PDMP data), pain in the first week after surgery (scale of 1-4: 1 = none, 2 = minimal, 3 = moderate, and 4 = severe), satisfaction with experience after surgery (scale of 0-10, with 10 being the highest satisfaction), and regret regarding undergoing surgery (scale of 1-5, with 1 being the highest level of regret). The second set of outcomes, which focused on opioid prescribing and dispensing, was calculated only for patients with both a discharge opioid prescription and a dispensed opioid prescription. Outcomes included total MMEs in the discharge opioid prescription, total MMEs in the dispensed opioid prescription, the occurrence of a dispensed opioid prescription exceeding a 5-day supply, days supplied in the dispensed opioid prescription, and the occurrence of at least 1 refill, defined as an additional opioid prescription filled after a dispensed opioid prescription but within 30 days of discharge from surgery or from hospitalization after surgery. We evaluated total MMEs both in the discharge and dispensed opioid prescription because these quantities could differ if, for example, pharmacists only partially filled a prescription.

### Statistical Analysis

We assigned patients to month based on the date of discharge and then aggregated outcomes to the monthly level. Using an interrupted time series analysis design, we fitted linear segmented regression models assessing for abrupt level or slope changes in monthly outcomes during February 2018.^21^ In a subgroup analysis, we repeated analyses among patients who were and were not opioid naive. In all models, we assessed for autocorrelation and used robust Newey-West errors with the appropriate number of lags. We conducted analyses from October 1, 2022, through February 28, 2023, using Stata, version 17.1/SE (StataCorp LLC), and used 2-sided hypothesis tests with α = .05.

A substantial proportion of patients was excluded at the last step of the sample selection algorithm owing to lack of complete data on the 3 patient-reported outcomes. We conducted several analyses to assess for any resulting selection bias. First, we compared the characteristics of patients included in the sample and those excluded at the last step. Second, among patients in the sample, we used linear regression to model pain scores as a function of each covariate, procedure type, and total MMEs prescribed. Subsequently, we calculated the expected pain score for patients excluded at the last step by multiplying the values of their covariates by the coefficients from the regression model. Finally, we identified patients who met all inclusion and exclusion criteria as of the second-to-last step in the sample selection algorithm. Among these patients, we compared the prevalence of missing data for patient-reported outcomes in the preintervention and postintervention periods, fitted segmented regression models for the 7 opioid prescribing and dispensing outcomes, and fitted segmented regression models assessing for level and slope changes in February 2018 in the monthly proportion of patients who were excluded owing to missing data on patient-reported outcomes.

## Results

### Sample

Among 17 600 patients meeting inclusion criteria, 11 329 met all exclusion criteria before the requirement for complete data on the 3 patient-reported outcomes. Of the 11 329 patients, 5284 (46.6%) were excluded owing to lack of these data, leaving 6045 patients (53.4%) (eFigure 1 in Supplement 1). Among these patients, the mean (SD) age was 48.7 (12.6) years, 2450 were male (40.5%), 3595 were female (59.5%), 131 were Hispanic (2.2%), 294 were non-Hispanic Black (4.9%), 5182 were non-Hispanic White (85.7%), 53 were of other race or ethnicity (0.9%; American Indian or Alaska Native, Asian, or Native Hawaiian or Pacific Islander), and 385 were of unknown race or ethnicity (6.4%). Race and ethnicity data were derived from the electronic health record and were typically based on patient self-report. The most common procedures were laparoscopic cholecystectomy (1663 patients [27.5%]), minor hernia repair (1624 patients [26.9%]), and laparoscopic appendectomy (676 patients [11.2%]). Characteristics of patients in the preintervention and postintervention periods were similar (body mass index ≥30 [calculated as weight in kilograms divided by height in meters squared], 653 [49.4%] vs 2479 [52.5%]; American Society of Anesthesiologists physical status class I, 178 [13.5%] vs 611 [12.9%]; elective surgery, 1033 [78.1%] vs 3613 [76.5%]; postoperative complications, 19 [1.4%] vs 89 [1.9%]; and opioid naive, 1090 [82.4%] vs 4021 [85.2%]) (Table 1).

**Table 1.** Sample Characteristics

Characteristic	Patients, No. (%)		
	All (N = 6045)	Preintervention period (n = 1323)^a^	Postintervention period (n = 4722)^a^
Age, mean (SD), y	48.7 (12.6)	48.7 (12.9)	48.7 (12.5)
Sex			
Male	2450 (40.5)	579 (43.8)	1871 (39.6)
Female	3595 (59.5)	744 (56.2)	2851 (60.4)
Race and ethnicity^b^			
Hispanic	131 (2.2)	31 (2.3)	100 (2.1)
Non-Hispanic Black	294 (4.9)	74 (5.6)	220 (4.7)
Non-Hispanic White	5182 (85.7)	1138 (86.0)	4044 (85.6)
Other	53 (0.9)	6 (0.5)	47 (1.0)
Unknown	385 (6.4)	74 (5.6)	311 (6.6)
Tobacco use	942 (15.6)	223 (16.9)	719 (15.2)
Cancer	267 (4.4)	48 (3.6)	219 (4.6)
Body mass index^c^			
<18.5	42 (0.7)	11 (0.8)	31 (0.7)
18.5-24.9	1041 (17.2)	242 (18.3)	799 (16.9)
25-29.9	1823 (30.2)	415 (31.4)	1408 (29.8)
≥30	3132 (51.8)	653 (49.4)	2479 (52.5)
Unknown	7 (0.1)	2 (0.2)	5 (0.1)
ASA physical status class^d^			
I	789 (13.1)	178 (13.5)	611 (12.9)
II	3932 (65.0)	875 (66.1)	3057 (64.7)
III	1281 (21.2)	261 (19.7)	1020 (21.6)
IV or V	38 (0.6)	7 (0.5)	31 (0.7)
Unknown	5 (0.1)	2 (0.2)	3 (0.1)
Surgical priority			
Elective	4646 (76.9)	1033 (78.1)	3613 (76.5)
Emergency or urgent	1399 (23.1)	290 (21.9)	1109 (23.5)
Inpatient	2872 (47.5)	649 (49.1)	2223 (47.1)
Procedure type			
Laparoscopic appendectomy	676 (11.2)	137 (10.4)	539 (11.4)
Laparoscopic cholecystectomy	1663 (27.5)	357 (27.0)	1306 (27.7)
Laparoscopic colectomy	195 (3.2)	41 (3.1)	154 (3.3)
Open colectomy	89 (1.5)	24 (1.8)	65 (1.4)
Minor hernia repair	1624 (26.9)	389 (29.4)	1235 (26.2)
Major hernia repair	222 (3.7)	51 (3.9)	171 (3.6)
Abdominal hysterectomy	232 (3.8)	49 (3.7)	183 (3.9)
Vaginal hysterectomy	319 (5.3)	63 (4.8)	256 (5.4)
Laparoscopic hysterectomy	637 (10.5)	119 (9.0)	518 (11.0)
Other	388 (6.4)	93 (7.0)	295 (6.2)
Postoperative complications	108 (1.8)	19 (1.4)	89 (1.9)
Opioid naive^e^	5111 (84.5)	1090 (82.4)	4021 (85.2)

Abbreviation: ASA, American Society of Anesthesiologists.

^a^ The preintervention period was defined as January 1, 2017, through January 31, 2018. The postintervention period was defined as February 1, 2018, through October 31, 2018.

^b^ Race and ethnicity data were derived from the electronic health record and were typically based on patient self-report. The other category included patients who were American Indian or Alaska Native, Asian, or Native Hawaiian or Pacific Islander.

^c^ Calculated as weight in kilograms divided by height in meters squared.

^d^ Class I, a person in good health; class II, a mild but well-managed or treated condition; class II, a serious condition that has an impact on a person’s overall health; class IV, a severe condition that is life threatening; and class V, a life-threatening condition that needs immediate surgery to increase survival odds.

^e^ Patients without opioid dispensing in the 120 days before discharge from surgery.

### Association Between Limit Implementation and Outcomes

**Figure 1.**
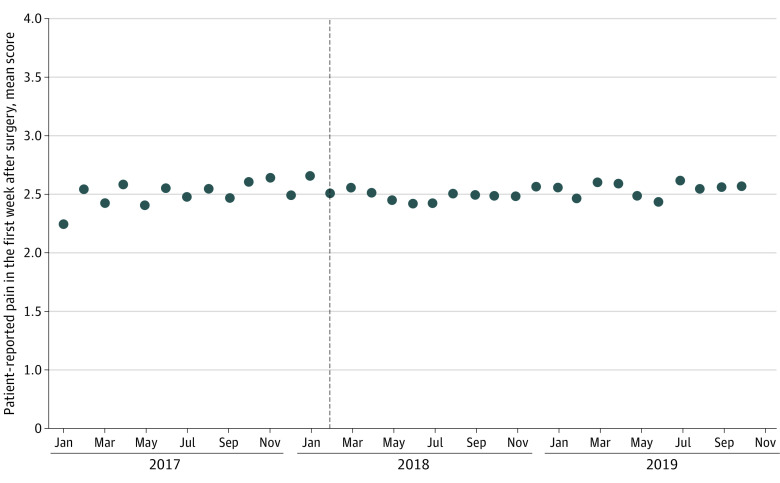
Association Between Implementation of the Blue Cross Blue Shield of Michigan Opioid Prescribing Limit and Monthly Mean Patient-Reported Pain in the Week After Surgery Pain was measured on a scale of 1 to 4 (1 = none, 2 = minimal, 3 = moderate, and 4 = severe). Dashed line indicates implementation of a Blue Cross Blue Shield of Michigan policy limiting the duration of new prescriptions (defined as those for patients lacking opioid dispensing in the prior 120 days) for short-acting opioids to a 5-day supply.

Table 2 displays coefficients from segmented regression models. Limit implementation was not associated with level or slope changes in the monthly proportion of patients with a discharge opioid prescription, but it was associated with a −6.3 percentage-point level (95% CI, −11.2 to −1.5 percentage-point level) decrease in the monthly proportion of patients with a dispensed opioid prescription. Limit implementation was not associated with level or slope changes in monthly mean patient-reported satisfaction or level of regret regarding undergoing surgery. Additionally, limit implementation was not associated with a level change in monthly mean patient-reported pain score in the first week after surgery and was associated with only a small level decrease in this score (−0.15 [95% CI, −0.26 to −0.03]), suggesting improved pain (Figure 1).

**Table 2.** Coefficients From Linear Segmented Regression Models Evaluating the Association Between Implementation of the Blue Cross Blue Shield of Michigan Opioid Prescribing Limit and Monthly Outcomes

Outcome	Intercept (95% CI)	Preintervention slope (95% CI)	Level change in February 2018 (95% CI)	Slope change in February 2018 (95% CI)
Patients with a discharge opioid prescription, %^a^	85.1 (77.3 to 92.8)	0.5 (−0.4 to 1.4)	−3.5 (−8.7 to 1.7)	−0.4 (−1.4 to 0.5)
Patients with a dispensed opioid prescription, %^b^	70.4 (66.1 to 74.6)	0.6 (0.1 to 1.0)	−6.3 (−11.2 to −1.5)	−0.2 (−0.8 to 0.3)
Mean pain score in the first week of surgery (scale of 1-4)	2.4 (2.3 to 2.5)	0.02 (0.00 to 0.03)	−0.15 (−0.26 to −0.03)	−0.01 (−0.03 to 0.00)
Mean satisfaction with care (scale of 0-10)	9.3 (9.2 to 9.4)	0.005 (−0.008 to 0.018)	−0.09 (−0.21 to 0.02)	−0.001 (−0.014 to 0.012)
Mean amount of regret regarding undergoing surgery (scale of 1-5)	4.9 (4.8 to 5.0)	−0.002 (−0.010 to 0.006)	0.01 (−0.05 to 0.07)	0.002 (−0.006 to 0.010)
Mean total morphine milligram equivalents in the discharge opioid prescription^c^	187.2 (177.3 to 197.0)	−4.1 (−5.3 to −2.9)	−22.3 (−32.8 to −11.9)	1.8 (0.5 to 3.1)
Mean total morphine milligram equivalents in the dispensed opioid prescription^c^	184.9 (164.9 to 204.8)	−3.5 (−6.0 to −1.1)	−26.1 (−40.9 to −11.3)	1.1 (−1.3 to 3.6)
Patients with dispensed opioid prescription >5-d supply, %^c^	42.7 (37.5 to 47.9)	−2.0 (−2.6 to −1.4)	−9.8 (−13.9 to −5.7)	1.6 (0.9 to 2.3)
Mean days supplied in discharge opioid prescription^c^	5.9 (5.4 to 6.3)	−0.11 (−0.16 to −0.06)	−0.8 (−1.2 to −0.4)	0.05 (−0.01 to 0.10)
Patients with at least 1 refill, %^c^	16.3 (11.8 to 20.9)	−0.7 (−1.3 to −0.1)	1.2 (−3.7 to 6.2)	0.63 (−0.03 to 1.30)

^a^ Defined as an opioid prescription prescribed at discharge from surgery.

^b^ Defined as an opioid prescription dispensed within 3 days of discharge from surgery (or within 3 days of discharge from hospitalization after surgery, as applicable).

^c^ These 5 outcomes were calculated only for patients with both a discharge and dispensed opioid prescription.

**Figure 2.**
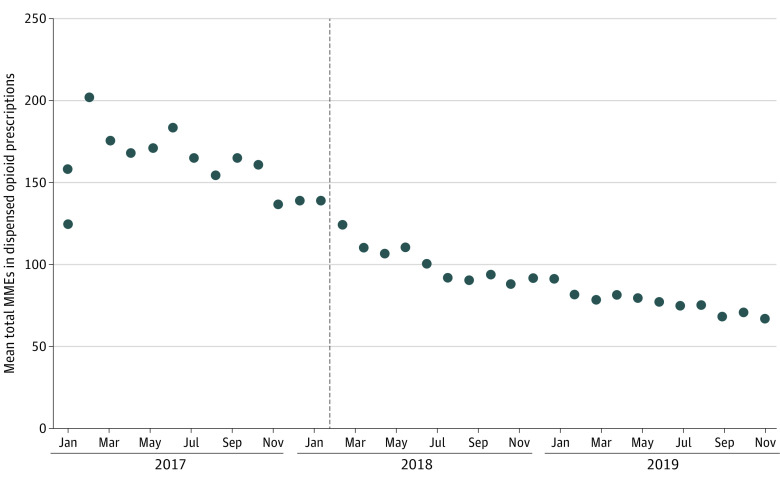
Association Between Implementation of the Blue Cross Blue Shield of Michigan Opioid Prescribing Limit and Mean Total Morphine Milligram Equivalents (MMEs) in Dispensed Opioid Prescriptions Dispensed opioid prescriptions were those filled within 3 days of discharge from surgery or within 3 days of discharge from hospitalization after surgery, as applicable. Dashed line indicates implementation of a Blue Cross Blue Shield of Michigan policy limiting the duration of new prescriptions (defined as those for patients lacking opioid dispensing in the prior 120 days) for short-acting opioids to a 5-day supply.

Among the 6045 patients, 1649 (27.3%) were excluded from the denominator of the remaining 5 outcomes, leaving 4396 patients (72.7%), all of whom had a discharge and dispensed opioid prescription. Among these patients, monthly mean total MMEs in the discharge opioid prescription decreased 4.1 MMEs (95% CI, −5.3 to −2.9 MMEs) per month before February 2018. Limit implementation was associated with a −22.3 (95% CI, −32.8 to −11.9) level decrease in this quantity, corresponding to approximately 3 pills containing 5 mg of oxycodone. Limit implementation was also associated with a −26.1 (95% CI, −40.9 to −11.3) level decrease in monthly mean total MMEs in the dispensed opioid prescription, corresponding to approximately 3.5 pills containing 5 mg of oxycodone (Figure 2). Among the 4396 patients, total MMEs in the discharge and dispensed opioid prescriptions differed for 269 patients (6.1%).

**Figure 3.**
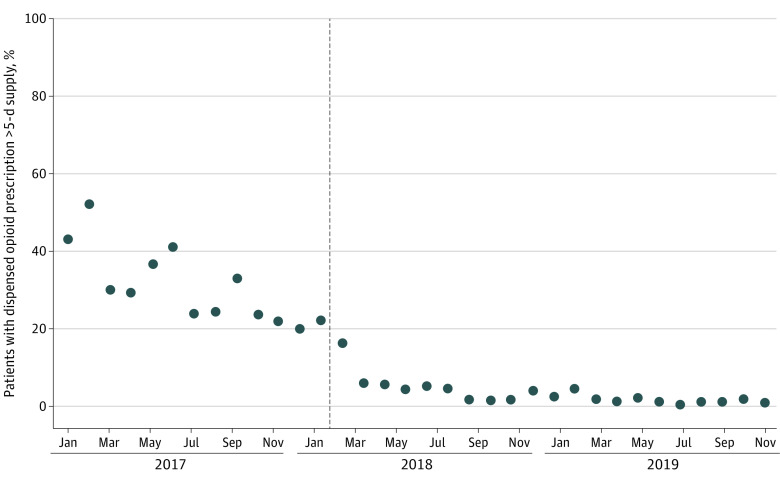
Association Between Implementation of the Blue Cross Blue Shield of Michigan Opioid Prescribing Limit and the Monthly Proportion of Patients With a Dispensed Opioid Prescription Exceeding a 5-Day Supply Dispensed opioid prescriptions were those filled within 3 days of discharge from surgery or within 3 days of discharge from hospitalization after surgery, as applicable. Dashed line indicates implementation of a Blue Cross Blue Shield of Michigan policy limiting the duration of new prescriptions (defined as those for patients lacking opioid dispensing in the prior 120 days) for short-acting opioids to a 5-day supply.

In January 2017 and October 2019, the beginning and end of the study period, monthly mean (SD) days supplied in dispensed opioid prescriptions were 5.3 (1.7) and 2.5 (1.0), respectively. Limit implementation was associated with a level decrease in this quantity of −0.8 days (95% CI, −1.2 to −0.4 days), representing a 13.6% decrease relative to the intercept. Limit implementation was also associated with a −9.8 percentage-point level (95% CI, −13.9 to −5.7 percentage-point level) decrease in the monthly proportion of patients with a dispensed opioid prescription exceeding a 5-day supply (Figure 3), but not with changes in the proportion of patients with at least 1 refill. eFigure 2 in Supplement 1 displays graphs for outcomes not depicted in Figure 1, Figure 2, and Figure 3, whereas eFigure 3 in Supplement 1 displays graphs for all outcomes with the fitted lines from segmented regression models.

### Subgroup Analyses

Among the 5111 opioid-naive patients (84.5%) in the 6045-patient sample, limit implementation was associated with changes similar to those in the overall analysis (eTable 1 and eFigure 4 in Supplement 1). For example, limit implementation was not associated with a level or slope change in mean pain scores even though it was associated with a 20.1 (95% CI, −30.6 to −9.6) level decrease in total MMEs in dispensed opioid prescriptions. Among the 934 non–opioid-naive patients (15.5%) in the sample, limit implementation was not associated with changes in the monthly proportion of patients with a dispensed opioid prescription, unlike in the overall analysis (eTable 2 in Supplement 1). Additionally, limit implementation was not associated with level changes in patient-reported outcomes, although it was associated with small slope changes.

Among 665 non–opioid-naive patients with BCBSM who had both a discharge and dispensed opioid prescription, limit implementation was associated with level decreases in monthly mean total MMEs in discharge opioid prescriptions (−31.4 MMEs [95% CI, −46.3 to −16.4 MMEs]), monthly mean total MMEs in dispensed opioid prescriptions (−33.7 MMEs [95% CI, −52.3 to −15.1 MMEs]), and monthly mean days supplied in dispensed opioid prescriptions (−1.1 days [95% CI, −1.8 to −0.3 days]). In contrast, limit implementation was not associated with level or slope changes in the monthly proportion of patients with at least 1 refill or the monthly proportion of patients with a dispensed opioid prescription exceeding a 5-day supply (eTable 2 in Supplement 1). However, the point estimate for the latter was negative (−11.8 percentage points [95% CI, −30.9 to 7.3 percentage points]).

### Additional Analyses to Assess for Selection Bias

Characteristics of the 6045 patients included in the sample and the 5284 patients excluded owing to lack of complete data on patient-reported outcomes were similar (body mass index ≥30, 3132 [51.8%] vs 2726 [51.6%]; American Society of Anesthesiologists physical status class I, 789 [13.1%] vs 677 [12.8%]; elective surgery, 4646 [76.9%] vs 4028 [76.2%]; and opioid naive, 5111 [84.5%] vs 4311 [81.6%]) (eTable 3 in Supplement 1). Among the 6045 patients included in the sample, the mean (SD) pain score was 2.5 (0.7), whereas the expected mean (SD) pain score among the 5284 excluded patients was 2.6 (0.2). Among patients who met all exclusion criteria before the requirement for complete data on the 3 patient-reported outcomes, the proportion excluded owing to missing data was similar in the preintervention (1051 of 2374 [44.3%]) and postintervention periods (4233 of 8955 [47.3%]). Among this same group of patients, conclusions for all but 1 of the 7 opioid prescribing and dispensing outcomes were unchanged compared with those of the main analysis. The coefficient for the level decrease in the proportion of patients with a dispensed opioid prescription became nonsignificant (−2.4%; 95% CI, −6.5% to 1.6%), although it was still negative, as in the main analysis. Finally, there was no level change (0.99%; 95% CI, −4.22% to 6.21%) in the monthly proportion of patients excluded owing to missing data on patient-reported outcomes, although there was a slope increase (1.1% per month; 95% CI, 0.3%-1.9%) (see eAppendix 4 in Supplement 1 for additional details).

## Discussion

In this analysis of data from adults undergoing common general surgical procedures across Michigan, implementation of a large commercial insurer’s 5-day opioid prescribing limit was not associated with worsened patient-reported outcomes, even though it was associated with reductions in the rate of opioid dispensing within 3 days of discharge from surgery, the amount of opioids prescribed and dispensed to patients, and the duration of dispensed opioid prescriptions. Findings suggest that limits—at least to the degree that they are applied to a patient population similar to ours—may be able to reduce opioid prescribing without worsening patient experience.

To our knowledge, this is the first large-scale study to evaluate the association between an insurer’s limit and both opioid prescribing and dispensing. Limit implementation was not associated with changes in the proportion of patients with a discharge opioid prescription, suggesting that prescribers did not respond to the limit by withholding opioid prescriptions. Despite this finding, limit implementation was associated with a decrease in the proportion of patients with a dispensed opioid prescription. One potential explanation is that BCBSM, pharmacists, or both rejected opioid prescriptions that were not compliant with the limit, leading to decreased dispensing.

Implementation of the limit was also associated with an immediate decline in the amount of opioids prescribed and dispensed. The magnitude of these declines—corresponding to approximately 3 to 3.5 pills—was arguably modest. One potential reason is that the amount of opioids prescribed and dispensed to patients had already decreased substantially by the time the limit was implemented, perhaps owing to factors such as a heightened focus on opioid stewardship after the release of national opioid prescribing guidelines in 2016.^22^ The modest decline in opioid prescribing and dispensing occurred even though BCBSM’s 5-day supply limit is more restrictive than most limits, which typically allow up to a 7-day supply of opioids,^7^ suggesting that most existing limits are set too high to substantially reduce opioid prescribing after surgery, a possibility supported by prior research.^13,14,15^

The lack of worsened patient-reported outcomes after limit implementation is consistent with a large body of literature evaluating other interventions to reduce surgical opioid prescribing.^23,24,25^ However, it would be incorrect to conclude that the limit was not associated with any unintended changes in patient care. In a subgroup analysis, limit implementation was associated with reductions in the amount of opioids prescribed and dispensed to non–opioid-naive patients, even though the limit targeted only opioid-naive patients. Findings highlight the importance of monitoring for spillover effects of opioid prescribing limits.^26^

When interpreting this study’s results, policymakers and insurers deciding whether to implement an opioid prescribing limit should consider an important caveat. The MSQC database predominantly captures patients undergoing common surgical procedures for which opioid needs are often modest.^27^ Findings may not generalize to procedures with higher opioid needs, such as neurosurgical procedures and certain orthopedic procedures (eg, total knee or hip replacement).^28^ For patients undergoing these procedures, limits could reduce opioid prescribing to a greater degree than among patients in this study, potentially increasing the risk of worsened patient-reported outcomes. Future studies should assess this possibility.

This study was enabled by our unique access to a statewide registry with data on patient-reported outcomes before and after a limit was implemented. However, there are challenges associated with creating and using these data. For example, owing to missing data on patient-reported outcomes, approximately half of patients who otherwise met inclusion and exclusion criteria were excluded. To maximize the utility of large-scale registries for quality improvement and research, funders should ensure sufficient resources for data collection. For example, they could provide funding for patient financial incentives to increase participation, funding to hire dedicated staff to follow up with patients for nonresponse, and funding to build the infrastructure to automate data collection in electronic health record systems.

### Limitations

This study has limitations. First, we could not identify an appropriate control group. We explored using other payer populations as a control group, such as privately insured MSQC patients not covered by BCBSM. However, preintervention trends were not parallel, suggesting these patients were not an ideal comparator. Second, estimates could be biased by the implementation of other opioid prescribing policies. For example, in June 2018, Michigan implemented a mandate for clinicians to query the PDMP before prescribing more than a 3-day supply of any controlled substance.^29^ Additionally, in July 2018, the state implemented a policy limiting the duration of opioid prescriptions for acute pain to a 7-day supply.^30^ However, our graphs did not demonstrate abrupt changes in opioid prescribing or dispensing outcomes during either June or July 2018. Moreover, the state limit was less restrictive than the 5-day BCBSM limit, suggesting the former was unlikely to affect opioid prescribing to BCBSM patients. Third, the exclusion of patients with missing data on patient-reported outcomes could introduce selection bias, although our additional analyses suggested that the magnitude of any bias was likely small.

## Conclusions

Implementation of a commercial insurer’s 5-day opioid prescribing limit was associated with reductions in opioid prescribing and dispensing but not with worsened patient-reported outcomes among adults undergoing common general surgical procedures in Michigan. Future research should determine whether these findings generalize to other limits and other surgical procedures, particularly invasive procedures with high opioid needs.

## Notes

Supplement 1. **eAppendix 1.** Procedures Included in the MSQC Database **eAppendix 2.** Details on Collection of Patient-Reported Outcome Data and Reasons for Missingness in These Data **eAppendix 3.** Rationale for Study Period **eAppendix 4.** Details on Analyses to Evaluate for Selection Bias Owing to Missing Data on Patient-Reported Outcomes **eFigure 1.** Sample Inclusion and Exclusion Criteria **eFigure 2.** Graphs for Other Outcomes **eFigure 3.** Graphs for All Ten Outcomes With Fitted Lines **eFigure 4.** Graphs of Outcomes for Opioid-Naïve Patients **eTable 1.** Subgroup Analyses for Opioid-Naïve Patients (n = 5,111) **eTable 2.** Subgroup Analyses for Non-Opioid Naïve Patients (n = 934) **eTable 3.** Characteristics of Patients Included in the Sample and Patients Excluded From the Sample Owing to Lack of Data on Patient-Reported Outcomes

Supplement 2.
Data Sharing Statement
