# Supplementary material for: Changes in Surgical Opioid Prescribing and Patient-Reported Outcomes After Implementation of an Insurer Opioid Prescribing Limit
Source: JAMA Health Forum. 2023 Oct 13;4(10):e233541. doi: 10.1001/jamahealthforum.2023.3541 (PMC10576220; doi:10.1001/jamahealthforum.2023.3541)
Supplement: Supplement 1. — eAppendix 1. Procedures Included in the MSQC Database eAppendix 2. Details on Collection of Patient-Reported Outcome Data and Reasons for Missingness in These Data eAppendix 3. Rationale for Study Period eAppendix 4. Details on Analyses to Evaluate for Selection Bias Owing to Missing Data on Patient-Reported Outcomes eFigure 1. Sample Inclusion and Exclusion Criteria eFigure 2. Graphs for Other Outcomes eFigure 3. Graphs for All Ten Outcomes With Fitted Lines eFigure 4. Graphs of Outcomes for Opioid-Naïve Patients eTable 1. Subgroup Analyses for Opioid-Naïve Patients (n = 5,111) eTable 2. Subgroup Analyses for Non-Opioid Naïve Patients (n = 934) eTable 3. Characteristics of Patients Included in the Sample and Patients Excluded From the Sample Owing to Lack of Data on Patient-Reported Outcomes [file jamahealthforum-e233541-s001.pdf]

Supplementary Online Content

Chua KP, Nguyen TD, Brummett CM, et al. Changes in surgical opioid prescribing and patient-reported outcomes after implementation of an insurer opioid prescribing limit. *JAMA Health Forum*. 2023;4(10):e233541. doi:10.1001/jamahealthforum.2023.3541

eAppendix 1. Procedures Included in the MSQC Database

eAppendix 2. Details on Collection of Patient-Reported Outcome Data and Reasons for Missingness in These Data

eAppendix 3. Rationale for Study Period

eAppendix 4. Details on Analyses to Evaluate for Selection Bias Owing to Missing Data on Patient-Reported Outcomes

eFigure 1. Sample Inclusion and Exclusion Criteria

eFigure 2. Graphs for Other Outcomes

eFigure 3. Graphs for All Ten Outcomes With Fitted Lines

eFigure 4. Graphs of Outcomes for Opioid-Naïve Patients

eTable 1. Subgroup Analyses for Opioid-Naïve Patients (n = 5,111)

eTable 2. Subgroup Analyses for Non-Opioid Naïve Patients (n = 934)

eTable 3. Characteristics of Patients Included in the Sample and Patients Excluded From the Sample Owing to Lack of Data on Patient-Reported Outcomes

This supplementary material has been provided by the authors to give readers additional information about their work.

© 2023 Chua KP et al. *JAMA Health Forum.*

eAppendix 1. Procedures included in the MSQC database

Listed below are the Current Procedural Terminology codes for surgical procedures included in the MSQC database.

0184T: Excision of rectal tumor, transanal endoscopic microsurgical approach (ie, TEMS), including muscularis propria (ie, full thickness)

0236T: Transluminal peripheral atherectomy, open or percutaneous, including radiological supervision and interpretation; abdominal aorta

0238T: Transluminal peripheral atherectomy, open or percutaneous, including radiological supervision and interpretation; iliac artery, each vessel

27590: Amputation, thigh, through femur, any level

27592: Amputation, thigh, through femur, any level; open, circular (guillotine)

27594: Amputation, thigh, through femur, any level; secondary closure or scar revision

27596: Amputation, thigh, through femur, any level; re-amputation

27598: Disarticulation at knee

27880: Amputation, leg, through tibia and fibula

27882: Amputation, leg, through tibia and fibula; open, circular (guillotine)

27884: Amputation, leg, through tibia and fibula; secondary closure or scar revision

27886: Amputation, leg, through tibia and fibula; re-amputation

34701: Endovascular repair of infrarenal aorta by deployment of an aorto-aortic tube endograft all endograft extension(s) placed in the aorta from the level of the renal arteries to the aortic bifurcation, and all angioplasty/stenting performed from the level of the renal arteries to the aortic bifurcation; for other than rupture (eg, for aneurysm, pseudoaneurysm, dissection, penetrating ulcer)

34702: Endovascular repair of infrarenal aorta by deployment of an aorto-aortic tube endograft all endograft extension(s) placed in the aorta from the level of the renal arteries to the aortic bifurcation, and all angioplasty/stenting performed from the level of the renal arteries to the aortic bifurcation; for rupture (eg, for aneurysm, pseudoaneurysm, dissection, penetrating ulcer, traumatic disruption)

34703: Endovascular repair of infrarenal aorta and/or iliac artery(ies) by deployment of an aorto-uni-iliac endograft all endograft extension(s) placed in the aorta from the level of the renal arteries to the iliac bifurcation, and all angioplasty/stenting performed from the level of the renal arteries to the iliac bifurcation; for other than rupture (eg, for aneurysm, pseudoaneurysm, dissection, penetrating ulcer)

34704: Endovascular repair of infrarenal aorta and/or iliac artery(ies) by deployment of an aorto-uni-iliac endograft including all endograft extension(s) placed in the aorta from the level of the renal arteries to the iliac bifurcation, and all angioplasty/stenting performed from the level of the renal arteries to the iliac bifurcation; for rupture (eg, for aneurysm, pseudoaneurysm, dissection, penetrating ulcer, traumatic disruption)

34705: Endovascular repair of infrarenal aorta and/or iliac artery(ies) by deployment of an aorto-bi-iliac endograft including all endograft extension(s) placed in the aorta from the level of the renal arteries to the iliac bifurcation, and all angioplasty/stenting performed from the level of the renal arteries to the iliac bifurcation; for other than rupture (eg, for aneurysm, pseudoaneurysm, dissection, penetrating ulcer)

34706: Endovascular repair of infrarenal aorta and/or iliac artery(ies) by deployment of an aorto-bi-iliac endograft including all endograft extension(s) placed in the aorta from the level of the renal arteries to the iliac bifurcation, and all angioplasty/stenting performed from the level of the renal arteries to the iliac bifurcation; for rupture (eg, for aneurysm, pseudoaneurysm, dissection, penetrating ulcer, traumatic disruption)

34707: Endovascular repair of iliac artery by deployment of an ilio-iliac tube endograft including pre-procedure sizing and device selection, all nonselective catheterization(s), all associated radiological supervision and interpretation, and all endograft extension(s) proximally to the aortic bifurcation and distally to the iliac bifurcation, and treatment zone angioplasty/stenting, when performed, unilateral; for other than rupture (eg, for aneurysm, pseudoaneurysm, dissection, arteriovenous malformation)

34708: Endovascular repair of iliac artery by deployment of an ilio-iliac tube endograft including and all endograft extension(s) proximally to the aortic bifurcation and distally to the iliac bifurcation, and treatment zone angioplasty/stenting, when performed, unilateral; for rupture (eg, for aneurysm, pseudoaneurysm, dissection, arteriovenous malformation, traumatic disruption)

34830: Open repair of infrarenal aortic aneurysm or dissection, plus repair of associated arterial trauma, following unsuccessful endovascular repair; tube prosthesis

34831: Open repair of infrarenal aortic aneurysm or dissection, plus repair of associated arterial trauma, following unsuccessful endovascular repair; aorto-bi-iliac prosthesis

© 2023 Chua KP et al. *JAMA Health Forum.*

34832: Open repair of infrarenal aortic aneurysm or dissection, plus repair of associated arterial trauma, following unsuccessful endovascular repair; aorto-bifemoral prosthesis
35081: Direct repair of aneurysm, pseudoaneurysm, or excision (partial or total) and graft insertion, with or without patch graft; for aneurysm, pseudoaneurysm, and associated occlusive disease, abdominal aorta

35082: Direct repair of aneurysm, pseudoaneurysm, or excision (partial or total) and graft insertion, with or without patch graft; for ruptured aneurysm, abdominal aorta
35091: Direct repair of aneurysm, pseudoaneurysm, or excision (partial or total) and graft insertion, with or without patch graft; for aneurysm, pseudoaneurysm, and associated occlusive disease, abdominal aorta involving visceral vessels (mesenteric, celiac, renal)
35092: Direct repair of aneurysm, pseudoaneurysm, or excision (partial or total) and graft insertion, with or without patch graft; for ruptured aneurysm, abdominal aorta involving visceral vessels (mesenteric, celiac, renal)
35102: Direct repair of aneurysm, pseudoaneurysm, or excision (partial or total) and graft insertion, with or without patch graft; for aneurysm, pseudoaneurysm, and associated occlusive disease, abdominal aorta involving iliac vessels (common, hypogastric, external)
35103: Direct repair of aneurysm, pseudoaneurysm, or excision (partial or total) and graft insertion, with or without patch graft; for ruptured aneurysm, abdominal aorta involving iliac vessels (common, hypogastric, external)
35131: Direct repair of aneurysm, pseudoaneurysm, or excision (partial or total) and graft insertion, with or without patch graft; for aneurysm, pseudoaneurysm, and associated occlusive disease, iliac artery (common, hypogastric, external)
35132: Direct repair of aneurysm, pseudoaneurysm, or excision (partial or total) and graft insertion, with or without patch graft; for ruptured aneurysm, iliac artery (common, hypogastric, external)
35141: Direct repair of aneurysm, pseudoaneurysm, or excision (partial or total) and graft insertion, with or without patch graft; for aneurysm, pseudoaneurysm, and associated occlusive disease, common femoral artery (profunda femoris, superficial femoral)
35142: Direct repair of aneurysm, pseudoaneurysm, or excision (partial or total) and graft insertion, with or without patch graft; for ruptured aneurysm, common femoral artery (profunda femoris, superficial femoral)
35151: Direct repair of aneurysm, pseudoaneurysm, or excision (partial or total) and graft insertion, with or without patch graft; for aneurysm, pseudoaneurysm, and associated occlusive disease, popliteal artery

35301: Thromboendarterectomy, including patch graft, if performed; carotid, vertebral, subclavian, by neck incision

35538: Bypass graft, with vein; aortobi-iliac

35539: Bypass graft, with vein; aortofemoral

35540: Bypass graft, with vein; aortobifemoral

35556: Bypass graft, with vein; femoral-popliteal

35558: Bypass graft, with vein; femoral-femoral

35565: Bypass graft, with vein; iliofemoral

35566: Bypass graft, with vein; femoral-anterior tibial, posterior tibial, peroneal artery or other distal vessels

35571: Bypass graft, with vein; popliteal-tibial, -peroneal artery or other distal vessels

35583: In-situ vein bypass; femoral-popliteal

35585: In-situ vein bypass; femoral-anterior tibial, posterior tibial, or peroneal artery

35587: In-situ vein bypass; popliteal-tibial, peroneal

35621: Bypass graft, with other than vein; axillary-femoral

35623: Bypass graft, with other than vein; axillary-popliteal or –tibial

35637: Bypass graft, with other than vein; aortoiliac

35638: Bypass graft, with other than vein; aortobi-iliac

35646: Bypass graft, with other than vein; aortobifemoral

35647: Bypass graft, with other than vein; aortofemoral

© 2023 Chua KP et al. *JAMA Health Forum.*

35654: Bypass graft, with other than vein; axillary-femoral-femoral

35656: Bypass graft, with other than vein; femoral-popliteal

35661: Bypass graft, with other than vein; femoral-femoral

35663: Bypass graft, with other than vein; ilioiliac

35665: Bypass graft, with other than vein; iliofemoral

35666: Bypass graft, with other than vein; femoral-anterior tibial, posterior tibial, or peroneal artery

35671: Bypass graft, with other than vein; popliteal-tibial or -peroneal artery

37220: Revascularization, endovascular, open or percutaneous, iliac artery, unilateral, initial vessel; with transluminal angioplasty
37221: Revascularization, endovascular, open or percutaneous, iliac artery, unilateral, initial vessel; with transluminal stent placement(s), includes angioplasty within the same vessel, when performed
37222: Revascularization, endovascular, open or percutaneous, iliac artery, each additional ipsilateral iliac vessel; with transluminal angioplasty (List separately in addition to code for primary procedure)
37223: Revascularization, endovascular, open or percutaneous, iliac artery, each additional ipsilateral iliac vessel; with transluminal stent placement(s), includes angioplasty within the same vessel, when performed (List separately in addition to code for primary procedure)

37224: Revascularization, endovascular, open or percutaneous, femoral, popliteal artery(s), unilateral; with transluminal angioplasty
37225: Revascularization, endovascular, open or percutaneous, femoral, popliteal artery(s), unilateral; with atherectomy, includes angioplasty within the same vessel, when performed
37226: Revascularization, endovascular, open or percutaneous, femoral, popliteal artery(s), unilateral; with transluminal stent placement(s), includes angioplasty within the same vessel, when performed
37227: Revascularization, endovascular, open or percutaneous, femoral, popliteal artery(s), unilateral; with transluminal stent placement(s) and atherectomy, includes angioplasty within the same vessel, when performed

37228: Revascularization, endovascular, open or percutaneous, tibial, peroneal artery, unilateral, initial vessel; with transluminal angioplasty
37229: Revascularization, endovascular, open or percutaneous, tibial, peroneal artery, unilateral, initial vessel; with atherectomy, includes angioplasty within the same vessel, when performed
37230: Revascularization, endovascular, open or percutaneous, tibial, peroneal artery, unilateral, initial vessel; with transluminal stent placement(s), includes angioplasty within the same vessel, when performed
37231: Revascularization, endovascular, open or percutaneous, tibial, peroneal artery, unilateral, initial vessel; with transluminal stent placement(s) and atherectomy, includes angioplasty within the same vessel, when performed
37232: Revascularization, endovascular, open or percutaneous, tibial/peroneal artery, unilateral, each additional vessel; with transluminal angioplasty (List separately in addition to code for primary procedure)
37233: Revascularization, endovascular, open or percutaneous, tibial/peroneal artery, unilateral, each additional vessel; with atherectomy, includes angioplasty within the same vessel, when performed (List separately in addition to code for primary procedure)
37234: Revascularization, endovascular, open or percutaneous, tibial/peroneal artery, unilateral, each additional vessel; with transluminal stent placement(s), includes angioplasty within the same vessel, when performed (List separately in addition to code for primary procedure)
37235: Revascularization, endovascular, open or percutaneous, tibial/peroneal artery, unilateral, each additional vessel; with transluminal stent placement(s) and atherectomy, includes angioplasty within the same vessel, when performed (List separately in addition to code for primary procedure)

38100: Splenectomy; total (separate procedure)

38101: Splenectomy; partial (separate procedure)

38102: Splenectomy; total, en bloc for extensive disease, in conjunction with other procedure (List in addition to code for primary procedure)

38115: Repair of ruptured spleen (splenorrhaphy) with or without partial splenectomy

38120: Laparoscopy, surgical, splenectomy

© 2023 Chua KP et al. *JAMA Health Forum.*

38129: Unlisted laparoscopy procedure, spleen

43100: Excision of lesion, esophagus, with primary repair; cervical approach

43101: Excision of lesion, esophagus, with primary repair; thoracic or abdominal approach

43107: Total or near total esophagectomy, without thoracotomy; with pharyngogastrostomy or cervical esophagogastrostomy, with or without pyloroplasty (transhiatal)

43108: Total or near total esophagectomy, without thoracotomy; with colon interposition or small intestine reconstruction, including intestine mobilization, preparation and anastomosis(es)

43112: Total or near total esophagectomy, with thoracotomy; with pharyngogastrostomy or cervical esophagogastrostomy, with or without pyloroplasty

43113: Total or near total esophagectomy, with thoracotomy; with colon interposition or small intestine reconstruction, including intestine mobilization, preparation, and anastomosis(es)

43116: Partial esophagectomy, cervical, with free intestinal graft, including microvascular anastomosis, obtaining the graft and intestinal reconstruction

43117: Partial esophagectomy, distal two-thirds, with thoracotomy and separate abdominal incision, with or without proximal gastrectomy; with thoracic esophagogastrostomy, with or without pyloroplasty (Ivor Lewis)

43118: Partial esophagectomy, distal two-thirds, with thoracotomy and separate abdominal incision, with or without proximal gastrectomy; with colon interposition or small intestine reconstruction, including intestine mobilization,

43121: Partial esophagectomy, distal two-thirds, with thoracotomy only, with or without proximal gastrectomy, with thoracic esophagogastrostomy, with or without pyloroplasty

43122: Partial esophagectomy, thoracoabdominal or abdominal approach, with or without proximal gastrectomy; with esophagogastrostomy, with or without pyloroplasty

43123: Partial esophagectomy, thoracoabdominal or abdominal approach, with or without proximal gastrectomy; with colon interposition or small intestine reconstruction, including intestine mobilization, preparation, and anastomosis(es)

43124: Total or partial esophagectomy, without reconstruction (any approach), with cervical

43130: Diverticulectomy of hypopharynx or esophagus, with or without myotomy; cervical approach

43135: Diverticulectomy of hypopharynx or esophagus, with or without myotomy; thoracic approach

43210: Esophagogastroduodenoscopy, flexible, transoral; diagnositc, including collection of specimen(s) by brushing or washing, when performed with esophagogastric fundoplasty, partial or complete, includes duodenoscopy when performed

43279: Laparoscopy, surgical, esophagomyotomy (Heller type), with fundoplasty, when performed

43280: Laparoscopy, surgical, esophagogastric fundoplasty (eg, Nissen, Toupet procedures)

43281: Laparoscopy, surgical, repair of paraesophageal hernia, includes fundoplasty, when performed; without implantation of mesh

43282: Laparoscopy, surgical, repair of paraesophageal hernia, includes fundoplasty, when performed; with implantation of mesh

43286: Esophagectomy, total or near total, with laparoscopic mobilization of the abdominal and mediastinal esophagus and proximal gastrectomy, with laparoscopic pyloric drainage procedure if performed, with open cervical pharyngogastrostomy or esophagogastrostomy (ie, laparoscopic transhiatal esophagectomy)

43287: Esophagectomy, distal two-thirds, with laparoscopic mobilization of the abdominal and lower mediastinal esophagus and proximal gastrectomy, with laparoscopic pyloric drainage procedure if performed, with separate thoracoscopic mobilization of the middle and upper mediastinal esophagus and thoracic esophagogastrostomy (ie, laparoscopic thoracoscopic esophagectomy, Ivor Lewis esophagectomy)

43288: Esophagectomy, total or near total, with thoracoscopic mobilization of the upper, middle, and lower mediastinal esophagus, with separate laparoscopic proximal gastrectomy, with laparoscopic pyloric drainage procedure if performed, with open cervical pharyngogastrostomy or esophagogastrostomy (ie, thoracoscopic, laparoscopic and cervical incision esophagectomy, McKeown esophagectomy, tri-incisional esophagectomy)

43289: Unlisted laparoscopy procedure, esophagus

43325: Esophagogastric fundoplasty, with fundic patch (Thal-Nissen procedure)

43327: Esophagogastric fundoplasty partial or complete; laparotomy

43328: Esophagogastric fundoplasty partial or complete; thoracotomy

43330: Esophagomyotomy (Heller type); abdominal approach

© 2023 Chua KP et al. *JAMA Health Forum.*

43331: Esophagomyotomy (Heller type); thoracic approach
43332: Repair, paraesophageal hiatal hernia (including fundoplication), via laparotomy, except neonatal; without implantation of mesh or other prosthesis
43333: Repair, paraesophageal hiatal hernia (including fundoplication), via laparotomy, except neonatal; with implantation of mesh or other prosthesis
43334: Repair, paraesophageal hiatal hernia (including fundoplication), via thoracotomy, except neonatal; without implantation of mesh or other prosthesis
43335: Repair, paraesophageal hiatal hernia (including fundoplication), via thoracotomy, except neonatal; with implantation of mesh or other prosthesis
43336: Repair, paraesophageal hiatal hernia, (including fundoplication), via thoracoabdominal incision, except neonatal; without implantation of mesh or other prosthesis
43337: Repair, paraesophageal hiatal hernia, (including fundoplication), via thoracoabdominal incision, except neonatal; with implantation of mesh or other prosthesis
43360: Gastrointestinal reconstruction for previous esophagectomy, for obstructing esophageal lesion or fistula, or for previous esophageal exclusion; with stomach, with or without pyloroplasty
43361: Gastrointestinal reconstruction for previous esophagectomy, for obstructing esophageal lesion or fistula, or for previous esophageal exclusion; with colon interposition or small intestine reconstruction, including intestine mobilization, preparation, and anastomosis(es)
43499: Unlisted procedure, esophagus
43500: Gastrotomy; with exploration or foreign body removal
43501: Gastrotomy; with suture repair of bleeding ulcer
43502: Gastrotomy; with suture repair of pre-existing esophagogastric laceration (eg, Mallory-Weiss)
43610: Excision, local; ulcer or benign tumor of stomach
43611: Excision, local; malignant tumor of stomach
43620: Gastrectomy, total; with esophagoenterostomy
43621: Gastrectomy, total; with Roux-en-Y reconstruction
43622: Gastrectomy, total; with formation of intestinal pouch, any type
43631: Gastrectomy, partial, distal; with gastroduodenostomy
43632: Gastrectomy, partial, distal; with gastrojejunostomy
43633: Gastrectomy, partial, distal; with Roux-en-Y reconstruction
43634: Gastrectomy, partial, distal; with formation of intestinal pouch
43659: Unlisted laparoscopy procedure, stomach
43820: Gastrojejunostomy; without vagotomy
43825: Gastrojejunostomy; with vagotomy, any type
43840: Gastrorrhaphy, suture of perforated duodenal or gastric ulcer, wound, or injury
43860: Revision of gastrojejunal anastomosis (gastrojejunostomy) with reconstruction, with or without partial gastrectomy or intestine resection; without vagotomy
43865: Revision of gastrojejunal anastomosis (gastrojejunostomy) with reconstruction, with or without partial gastrectomy or intestine resection; with vagotomy
43999: Unlisted procedure, stomach
44005: Enterolysis (freeing of intestinal adhesion) (separate procedure)
44020: Enterotomy, small intestine, other than duodenum; for exploration, biopsy(s), or foreign body removal
44021: Enterotomy, small intestine, other than duodenum; for decompression (eg, Baker tube)
44050: Reduction of volvulus, intussusception, internal hernia, by laparotomy
44055: Correction of malrotation by lysis of duodenal bands and/or reduction of midgut volvulus (eg, Ladd procedure)
44120: Enterectomy, resection of small intestine; single resection and anastomosis

© 2023 Chua KP et al. *JAMA Health Forum.*

44125: Enterectomy, resection of small intestine; with enterostomy
44130: Enteroenterostomy, anastomosis of intestine, with or without cutaneous enterostomy (separate procedure)
44140: Colectomy, partial; with anastomosis
44141: Colectomy, partial; with skin level cecostomy or colostomy
44143: Colectomy, partial; with end colostomy and closure of distal segment (Hartmann type procedure)
44144: Colectomy, partial; with resection, with colostomy or ileostomy and creation of mucofistula
44145: Colectomy, partial; with coloproctostomy (low pelvic anastomosis)
44146: Colectomy, partial; with coloproctostomy (low pelvic anastomosis), with colostomy
44147: Colectomy, partial; abdominal and transanal approach; with anastomosis
44150: Colectomy, total, abdominal, without proctectomy; with ileostomy or ileoproctostomy
44151: Colectomy, total, abdominal, without proctectomy; with continent ileostomy
44155: Colectomy, total, abdominal, with proctectomy; with ileostomy
44156: Colectomy, total, abdominal, with proctectomy; with continent ileostomy
44157: Colectomy, total, abdominal, with proctectomy; with ileoanal anastomosis, includes loop ileostomy, and rectal mucosectomy, when performed
44158: Colectomy, total, abdominal, with proctectomy; with ileoanal anastomosis, creation of ileal reservoir (S or J), includes loop ileostomy, and rectal mucosectomy, when performed
44160: Colectomy, partial, with removal of terminal ileum with ileocolostomy
44180: Laparoscopy, surgical, enterolysis (freeing of intestinal adhesion) (separate procedure)
44187: Laparoscopy, surgical; ileostomy or jejunostomy, non-tube
44188: Laparoscopy, surgical, colostomy or skin level cecostomy
44202: Laparoscopy, surgical; enterectomy, resection of small intestine, single resection and anastomosis
44204: Laparoscopy, surgical; colectomy, partial, with anastomosis
44205: Laparoscopy, surgical; colectomy, partial, with removal of terminal ileum with ileocolostomy
44206: Laparoscopy, surgical; colectomy, partial, with end colostomy and closure of distal segment (Hartmann type procedure)
44207: Laparoscopy, surgical; colectomy, partial, with anastomosis, with coloproctostomy (low pelvic anastomosis)
44208: Laparoscopy, surgical; colectomy, partial, with anastomosis, with coloproctostomy (low pelvic anastomosis) with colostomy
44210: Laparoscopy, surgical; colectomy, total, abdominal, without proctectomy, with ileostomy or ileoproctostomy
44211: Laparoscopy, surgical; colectomy, total, abdominal, with proctectomy, with ileoanal anastomosis, creation of ileal reservoir (S or J), with loop ileostomy, includes rectal mucosectomy, when performed
44212: Laparoscopy, surgical; colectomy, total, abdominal, with proctectomy, with ileostomy
44227: Laparoscopy, surgical, closure of enterostomy, large or small intestine, with resection and anastomosis
44238: Unlisted laparoscopy procedure, large or small intestine (except rectum)
44310: Ileostomy or jejunostomy, non-tube
44312: Revision of ileostomy; simple (release of superficial scar) (separate procedure)
44314: Revision of ileostomy, complicated (reconstruction in-depth) (separate procedure)
44320: Colostomy or skin level cecostomy
44322: Colostomy or skin level cecostomy; with multiple biopsies (e.g. for congenital megacolon) (separate procedure)

© 2023 Chua KP et al. *JAMA Health Forum.*

44340: Revision of colostomy; simple (release of superficial scar) (separate procedure)
44345: Revision of colostomy; complicated (reconstruction in-depth) (separate procedure)
44346: Revision of colostomy; with repair of paracolostomy hernia (separate procedure)
44602: Suture of small intestine (enterorrhaphy) for perforated ulcer, diverticulum, wound, injury or rupture; single perforation
44603: Suture of small intestine (enterorrhaphy) for perforated ulcer, diverticulum, wound, injury or rupture; multiple perforations
44604: Suture of large intestine (colorrhaphy) for perforated ulcer, diverticulum, wound, injury or rupture (single or multiple perforations); without colostomy
44605: Suture of large intestine (colorrhaphy) for perforated ulcer, diverticulum, wound, injury or rupture (single or multiple perforations); with colostomy
44615: Intestinal stricturoplasty (enterotomy and enterorrhaphy) with or without dilation, for intestinal obstruction
44620: Closure of enterostomy, large or small intestine;
44625: Closure of enterostomy, large or small intestine; with resection and anastomosis other than colorectal
44626: Closure of enterostomy, large or small intestine; with resection and colorectal anastomosis (eg, closure of Hartmann type procedure)
44640: Closure of intestinal cutaneous fistula
44650: Closure of enteroenteric or enterocolic fistula
44660: Closure of enterovesical fistula; w/out intestinal or bladder resection
44661: Closure of enterovesical fistula; with intestine and/or bladder resection
44799: Unlisted procedure, small intestine
44800: Excision of Meckel's diverticulum (diverticulectomy) or omphalomesenteric duct
44950: Appendectomy
44960: Appendectomy; for ruptured appendix with abscess or generalized peritonitis
44970: Laparoscopy, surgical, appendectomy
45110: Proctectomy; complete, combined abdominoperineal, with colostomy
45111: Proctectomy; partial resection of rectum, transabdominal approach
45112: Proctectomy, combined abdominoperineal, pull-through procedure (eg, colo-anal anastomosis)
45113: Proctectomy, partial, with rectal mucosectomy, ileoanal anastomosis, creation of ileal reservoir (S or J), with or without loop ileostomy
45114: Proctectomy, partial, with anastomosis; abdominal and transsacral approach
45116: Proctectomy, partial, with anastomosis; transsacral approach only (Kraske type)
45119: Proctectomy, combined abdominoperineal pull-through procedure (eg, colo-anal anastomosis), with creation of colonic reservoir (eg, J-pouch), with diverting enterostomy when performed
45120: Proctectomy, complete (for congenital megacolon), abdominal and perineal approach; with pull-through procedure and anastomosis (eg, Swenson, Duhamel, or Soave type operation)
45121: Proctectomy, complete (for congenital megacolon), abdominal and perineal approach; with subtotal or total colectomy, with multiple biopsies
45123: Proctectomy, partial, without anastomosis, perineal approach
45126: Pelvic Exenteration for Colorectal Malignancy, with Protectomy (w/ or w/out colostomy), with removal of bladder and ureteral transplantations, and/or Hysterectomy, or cervicectomy, w/ or w/out removal of tubes(s), and w/ or w/out removal of ovary(s),
45130: Excision of rectal procidentia, with anastomosis; perineal approach
45135: Excision of rectal procidentia, with anastomosis; abdominal and perineal approach
45136: Excision of ileoanal reservoir with ileostomy
45160: Excision of rectal tumor by proctotomy, transsacral or transcoccygeal approach
© 2023 Chua KP et al. *JAMA Health Forum.*

45171: Excision of rectal tumor, transanal approach; not including muscularis propria (ie, partial thickness)

45172: Excision of rectal tumor, transanal approach; including muscularis propria (ie, full thickness)

45395: Laparoscopy, surgical; proctectomy, complete, combined abdominoperineal, with colostomy

45397: Laparoscopy, surgical; proctectomy, combined abdominoperineal pull-through procedure (eg, colo-anal anastomosis), with creation of colonic reservoir (eg, J-pouch), with diverting enterostomy, when performed

45399: Unlisted procedure, colon

45400: Laparoscopy, surgical proctopexy (for prolapse)

45402: Laparoscopy, surgical proctopexy (for prolapse), with sigmoid resection

45540: Proctopexy (e.g. for prolapse); abdominal approach

45550: Proctopexy (e.g. for prolapse); with sigmoid resection, abdominal approach

47120: Hepatectomy, resection of liver; partial lobectomy

47122: Hepatectomy, resection of liver; trisegmentectomy

47125: Hepatectomy, resection of liver; total left lobectomy

47130: Hepatectomy, resection of liver; total right lobectomy

47379: Unlisted laparoscopic procedure, liver

47562: Laparoscopy, surgical; cholecystectomy

47563: Laparoscopy, surgical; cholecystectomy with cholangiography

47564: Laparoscopy, surgical; cholecystectomy with exploration of common duct

47600: Cholecystectomy

47605: Cholecystectomy; with cholangiography

47610: Cholecystectomy with exploration of common duct

47612: Cholecystectomy with exploration of common duct, with Choledochoenterostomy

47620: Cholecystectomy with exploration of common duct, with Transduodenal Spincterotomy or Sphincteroplasty, w/ or w/out Cholangiography

47760: Anastomosis, of extrahepatic biliary ducts and gastrointestinal tract

47765: Anastomosis, of intrahepatic ducts and gastrointestinal tract

47780: Anastomosis, Roux-en-Y, of extrahepatic biliary ducts and gastrointestinal tract

47785: Anastomosis, Roux-en-Y, of intrahepatic biliary ducts and gastrointestinal tract

48105: Resection or debridement of pancreas and peripancreatic tissue for acute necrotizing pancreatitis

48120: Excision of lesion of pancreas (eg, cyst, adenoma)

48140: Pancreatectomy, distal subtotal, with or without splenectomy; without pancreaticojejunostomy

48145: Pancreatectomy, distal subtotal, with or without splenectomy; with pancreaticojejunostomy

48146: Pancreatectomy, distal, near-total with preservation of duodenum (Child-type procedure)

48148: Excision of ampulla of Vater

48150: Pancreatectomy, proximal subtotal with total duodenectomy, partial gastrectomy, choledochoenterostomy and gastrojejunostomy (Whipple-type procedure); with pancreatojejunostomy

48152: Pancreatectomy, proximal subtotal with total duodenectomy, partial gastrectomy, choledochoenterostomy and gastrojejunostomy (Whipple-type procedure); without pancreatojejunostomy

© 2023 Chua KP et al. *JAMA Health Forum.*

48153: Pancreatectomy, proximal subtotal with near-total duodenectomy, choledochoenterostomy and duodenojejunostomy (pylorus-sparing, Whipple-type procedure); with pancreatojejunostomy

48154: Pancreatectomy, proximal subtotal with near-total duodenectomy, choledochoenterostomy and duodenojejunostomy (pylorus-sparing, Whipple-type procedure); without pancreatojejunostomy

48155: Pancreatectomy, total

48999: Unlisted procedure, pancreas

49505: Repair initial inguinal hernia, age 5 years or older; reducible

49507: Repair initial inguinal hernia, age 5 years or older; incarcerated or strangulated

49520: Repair recurrent inguinal hernia, any age; reducible

49521: Repair recurrent inguinal hernia, any age; incarcerated or strangulated

49525: Repair inguinal hernia, sliding, any age

49550: Repair initial femoral hernia, any age; reducible

49553: Repair initial femoral hernia, any age; incarcerated or strangulated

49555: Repair recurrent femoral hernia; reducible

49557: Repair recurrent femoral hernia; incarcerated or strangulated

49560: Repair initial incisional or ventral hernia; reducible

49561: Repair initial incisional or ventral hernia; incarcerated or strangulated

49565: Repair recurrent incisional or ventral hernia; reducible

49566: Repair recurrent incisional or ventral hernia; incarcerated or strangulated

49570: Repair epigastric hernia; reducible

49572: Repair epigastric hernia; incarcerated or strangulated

49585: Repair umbilical hernia, age 5 years or older; reducible

49587: Repair umbilical hernia, age 5 years or older; incarcerated or strangulated

49590: Repair spigelian hernia

49650: Laparoscopy, surgical; repair initial inguinal hernia

49651: Laparoscopy, surgical; repair recurrent inguinal hernia

49652: Laparoscopy, surgical, repair, ventral, umbilical, spigelian or epigastric hernia (includes mesh insertion, when performed); reducible

49653: Laparoscopy, surgical, repair, ventral, umbilical, spigelian or epigastric hernia (includes mesh insertion, when performed); incarcerated or strangulated

49654: Laparoscopy, surgical, repair, incisional hernia (includes mesh insertion, when performed); reducible

49655: Laparoscopy, surgical, repair, incisional hernia (includes mesh insertion, when performed); incarcerated or strangulated

49656: Laparoscopy, surgical, repair, recurrent incisional hernia (includes mesh insertion, when performed); reducible

49657: Laparoscopy, surgical, repair, recurrent incisional hernia (includes mesh insertion, when performed); incarcerated or strangulated

49659: Unlisted laparoscopy procedure, hernioplasty, herniorrhaphy, herniotomy

58150: Total abdominal hysterectomy (corpus and cervix), with or without removal of tube(s), with or without removal of ovary(s)

58152: Total abdominal hysterectomy (corpus and cervix), with or without removal of tube(s), with or without removal of ovary(s); with colpo-urethrocystopexy (eg, Marshall-Marchetti-Krantz, Burch)

58180: Supracervical abdominal hysterectomy (subtotal hysterectomy), with or without removal of tube(s), with or without removal of ovary(s)

© 2023 Chua KP et al. *JAMA Health Forum.*

58200: Total abdominal hysterectomy, including partial vaginectomy, with para-aortic and pelvic lymph node sampling, with or without removal of tube(s), with or without removal of ovary(s)
58210: Radical abdominal hysterectomy, with bilateral total pelvic lymphadenectomy and para-aortic lymph node sampling (biopsy), with or without removal of tube(s), with or without removal of ovary(s)
58240: Pelvic exenteration for gynecologic malignancy, with total abdominal hysterectomy or cervicectomy, with or without removal of tube(s), with or without removal of ovary(s), with removal of bladder and ureteral transplantations, and/or abdominoperineal resection of rectum and colon and colostomy, or any combination thereof

58260: Vaginal hysterectomy, for uterus 250 g or less

58262: Vaginal hysterectomy, for uterus 250 g or less; with removal of tube(s), and/or ovary(s)

58263: Vaginal hysterectomy, for uterus 250 g or less; with removal of tube(s), and/or ovary(s), with repair of enterocele

58267: Vaginal hysterectomy, for uterus 250 g or less; with colpo-urethrocystopexy (Marshall-Marchetti-Krantz type, Pereyra type) with or without endoscopic control

58270: Vaginal hysterectomy, for uterus 250 g or less; with repair of enterocele

58275: Vaginal hysterectomy, with total or partial vaginectomy;

58280: Vaginal hysterectomy, with total or partial vaginectomy; with repair of enterocele

58285: Vaginal hysterectomy, radical (Schauta type operation)

58290: Vaginal hysterectomy, for uterus greater than 250 g

58291: Vaginal hysterectomy, for uterus greater than 250 g; with removal of tube(s) and/or ovary(s)

58292: Vaginal hysterectomy, for uterus greater than 250 g; with removal of tube(s) and/or ovary(s), with repair of enterocele

58293: Vaginal hysterectomy, for uterus greater than 250 g; with colpo-urethrocystopexy (Marshall- Marchetti-Krantz type, Pereyra type) with or without endoscopic control

58294: Vaginal hysterectomy, for uterus greater than 250 g; with repair of enterocele

58541: Laparoscopy, surgical, supracervical hysterectomy, for uterus 250 g or less;

58542: Laparoscopy, surgical, supracervical hysterectomy, for uterus 250 g or less; with removal of tube(s) and/or ovary(s)

58543: Laparoscopy, surgical, supracervical hysterectomy, for uterus greater than 250 g;

58544: Laparoscopy, surgical, supracervical hysterectomy, for uterus greater than 250 g; with removal of tube(s) and/or ovary(s)
58548: Laparoscopy, surgical, with radical hysterectomy, with bilateral total pelvic lymphadenectomy and para-aortic lymph node sampling (biopsy), with removal of tube(s) and ovary(s), if performed

58550: Laparoscopy, surgical, with vaginal hysterectomy, for uterus 250 g or less

58552: Laparoscopy, surgical, with vaginal hysterectomy, for uterus 250 g or less; with removal of tube(s) and/or ovary(s)

58553: Laparoscopy, surgical, with vaginal hysterectomy, for uterus greater than 250 g

58554: Laparoscopy, surgical, with vaginal hysterectomy, for uterus greater than 250 g; with removal of tube(s) and/or ovary(s)

58570: Laparoscopy, surgical, with total hysterectomy, for uterus 250 g or less

58571: Laparoscopy, surgical, with total hysterectomy, for uterus 250 g or less; with removal of tube(s) and/or ovary(s)

58572: Laparoscopy, surgical, with total hysterectomy, for uterus greater than 250 g

58573: Laparoscopy, surgical, with total hysterectomy, for uterus greater than 250 g; with removal of tube(s) and/or ovary(s)
58575: Laparoscopy, surgical, total hysterectomy for resection of malignancy (tumor debulking), with omentectomy including salpingo-oophorectomy, unilateral or bilateral, when performed
58951: Resection (initial) of ovarian, tubal or primary peritoneal malignancy with bilateral salpingo- oophorectomy and omentectomy; with total abdominal hysterectomy, pelvic and limited para-aortic lymphadenectomy

58953: Bilateral salpingo-oophorectomy with omentectomy, total abdominal hysterectomy and radical dissection for debulking

© 2023 Chua KP et al. *JAMA Health Forum.*

58954: Bilateral salpingo-oophorectomy with omentectomy, total abdominal hysterectomy and radical dissection for debulking; with pelvic lymphadenectomy and limited para-aortic lymphadenectomy

58956: Bilateral salpingo-oophorectomy with total omentectomy, total abdominal hysterectomy for malignancy

59525: Subtotal or total hysterectomy after cesarean delivery (List separately in addition to code for primary procedure)

60210: Partial thyroid lobectomy, unilateral; with or without isthmusectomy

60212: Partial thyroid lobectomy, unilateral; with contralateral subtotal lobectomy, including isthmusectomy

60220: Total thyroid lobectomy, unilateral; with or without isthmusectomy

60225: Total thyroid lobectomy, unilateral; with contralateral subtotal lobectomy, including isthmusectomy

60240: Thyroidectomy, total or complete

60252: Thyroidectomy, total or subtotal for malignancy; with limited neck dissection

60254: Thyroidectomy, total or subtotal for malignancy; with radical neck dissection

60260: Thyroidectomy, removal of all remaining thyroid tissue following previous removal of a portion of thyroid

60270: Thyroidectomy, including substernal thyroid; sternal split or transthoracic approach

60271: Thyroidectomy, including substernal thyroid; cervical approach

60540: Adrenalectomy, partial or complete, or exploration of adrenal gland with or without biopsy, transabdominal, lumbar or dorsal (separate procedure);
60545: Adrenalectomy, partial or complete, or exploration of adrenal gland with or without biopsy, transabdominal, lumbar or dorsal (separate procedure); with excision of adjacent retroperitoneal tumor

60650: Laparoscopy, surgical, with adrenalectomy, partial or complete, or exploration of adrenal gland with or without biopsy, transabdominal, lumbar or dorsal

© 2023 Chua KP et al. *JAMA Health Forum.*

eAppendix 2. Details on collection of patient-reported outcome data, reasons for missingness in these data, and analyses to evaluate for selection bias owing to missing data

In 2017, approximately half of MSQC hospitals began collecting patient-reported outcome data as part of a pilot project. In 2018, there was a sharp increase in the number of hospitals collecting these data. By 2019, all MSQC hospitals were collecting these data. Each MSQC hospital uses the same survey instrument to collect patient-reported outcome data, and all send the surveys at the same time (between 30-90 days after discharge).

In our study, 11,329 patients had met all inclusion and exclusion criteria by the second-to-last step in **eFigure 1.** Of these 11,329 patients, 5,284 (47.6%) were excluded at the last step owing to lack of complete data for the 3-paitent reported outcomes. The reasons for this missingness are three-fold. First, owing to resource limitations, not every patient during the study period was sent a survey assessing patient-reported outcomes. Second, some patients sent the survey did not respond. Third, 292 patients were excluded because they only responded to 1 or 2 of the questions assessing patient-reported outcomes, not all 3. Unfortunately, the study team, which includes the director of the MSQC initiative (Dr. Michael Englesbe), does not have information on how many surveys each hospital sent out, so we cannot determine the degree to which missing data on the 3 patient-reported outcomes was driven by the first reason above (surveys not being sent out to all patients) versus survey non-response.

© 2023 Chua KP et al. *JAMA Health Forum.*

eAppendix 3. Rationale for study period

In 2020, we submitted a request for MSQC records from January 2017 through October 2019 to be linked to the Michigan prescription drug monitoring program database via the University of Michigan's honest broker office. The resulting linked database formed the basis for our sample. Although we have since submitted a request for MSQC records from November 2019 onwards to be linked to the PDMP, technical limitations preclude combining different extracts.

© 2023 Chua KP et al. *JAMA Health Forum.*

eAppendix 4. Details on analyses to evaluate for selection bias owing to missing data on patient-reported outcomes

We conducted several analyses to assess the possibility of selection bias owing to missing data.

1) We compared the characteristics of the 6,045 patients included in the sample and the 5,284 patients excluded at the last step of the sample selection algorithm owing to lack of complete data on the 3 patient-reported outcomes. As shown in **eTable 1,** differences were sometimes statistically significant, but the absolute magnitude of differences was small.

2) Among the 6,045 patients included in our sample, we used linear regression to model pain scores as a function of each of the characteristics in Table 1, procedure type, and total MME prescribed (if no discharge opioid prescription occurred, we set total MMEs to 0). For each of the 5,284 patients excluded at the last step owing to lack of complete data on the 3 patient-reported outcomes, we calculated the predicted pain score by multiplying the coefficients from the regression model and the observed value of the covariates for the excluded patient. Among the 6,045 patients in our sample, the mean pain score was 2.5, while the predicted mean pain score among the 5,284 excluded patients was 2.6. We acknowledge that this analysis makes the strong assumption that pain scores are perfectly predicted by observed characteristics, procedure type, and total MME prescribed. Despite this, we believe it underscores the fact that included and excluded patients were largely similar on observed characteristics.

3) We assessed for differences in the prevalence of missing data for patient-reported outcomes in the pre-intervention and post-intervention period. There were 2,374 patients in the pre-intervention period who met all inclusion and exclusion criteria as of the second-to-last step in our sample selection algorithm. Of these, 1,051 (44.3%) were excluded owing to lack of complete data on the 3 patient-reported outcomes. Among the 39 hospitals, this proportion ranged from 0-100%; the median was 46.9% and the 25th-75th percentile was 15.4%-65.4%. There were 8,955 patients in the post-intervention period who met all inclusion and exclusion criteria as of the second-to-last step in our sample selection algorithm. Of these, 4,233 (47.3%) were excluded owing to lack of complete data on the 3 patient-reported outcomes. Among the 39 hospitals, this proportion ranged from 5.3%-96.9%; the median was 49.0% and the 25th-75th percentile was 32.5%-60.6%. The fact that the proportion of patients excluded owing to lack of complete data on the 3 patient-reported outcomes was only slightly lower in the pre-intervention period (44.3%) compared with the post-intervention period (47.3%) suggests that the magnitude of any selection bias owing to missing data likely did not change substantially over time.

4) Among the 11,329 patients who met all inclusion and exclusion criteria as of the second to last step in our sample selection algorithm, we conducted interrupted time series analyses for the 7 opioid prescribing and dispensing outcomes. Conclusions were unchanged, except that the coefficient for the level decrease in the proportion of patients with a dispensed opioid prescription became non-significant (-2.4%, 95% CI: -6.5%, 1.6%), although it was still negative, as in the main analysis (see **Table** below).

5) Among the 11,329 patients, we conducted an interrupted time series analysis evaluating for level and slope changes in February 2018 in the monthly proportion of patients who were excluded because they lacked complete data on the 3 patient-reported outcomes. There was no level change in this outcome (1.0%, 95% CI: -4.2, 6.2%), although there was a slope increase (1.1% per month, 95% CI: 0.3%, 1.9%).

© 2023 Chua KP et al. *JAMA Health Forum.*

Collectively, these analyses suggest that selection bias owing to missing patient-reported outcome data is possible, but they do not support the notion that this bias was likely to be large enough to alter the main conclusions of this paper.

Table. Outcomes among the 6,045 patients included in the sample and the 5,284 patients excluded owing to incomplete data for the 3 patient-reported outcomes (total n = 11,329)

Outcome	Intercept [95% CI]	Pre-intervention slope [95% CI]	Level change in February 2018 [95%CI]	Slope change in February 2018 [95% CI]
% patients with a discharge opioid prescription	87.7% [86.4%, 88.9%]	0.07% [-0.08%, 0.2%]	-0.4% [-2.2%, 1.3%]	-0.1% [-0.3%, 0.07%]
% patients with a dispensed opioid prescription	72.3% [68.3%, 76.4%]	0.2% [-0.3%, 0.7%]	-2.4% [-6.5%, 1.6%]	-0.05% [-0.6%, 0.5%]
Mean total morphine milligram equivalents in the discharge opioid prescription	188.3 [173.3, 203.3]	-4.1 [-5.9, -2.3]	-23.4 [-34.2, -12.5]	2.0 [0.1, 3.8]
Mean total morphine milligram equivalents in the dispensed opioid prescription	185.9 [172.5, 199.4]	-3.6 [-5.4, -1.9]	-26.4 [-38.3, -14.5]	1.5 [-0.3, 3.3]
% patients with dispensed opioid prescription > 5-day supply	37.6% [31.2%, 43.9%]	-1.3% [-2.0%, -0.6%]	-14.4% [-18.8%, -10.0%]	1.0% [0.3%, 1.7%]
Mean days supplied in discharge opioid prescription	5.5 [5.2, 5.9]	-0.07 [-0.1, -0.03]	-1 [-1.3, -0.7]	0.01 [-0.03, 0.06]
% patients with at least one refill	14.9% [12.2%, 17.6%]	-0.4% [-0.8%, -0.03%]	0.5% [-2.6%, 3.6%]	0.3% [-0.1%, 0.8%]
% patients excluded owing to lack of complete data on the 3 patient-reported outcomes	49.4% [42.3%, 56.5%]	-0.7% [-1.4%, 0.1%]	1.0% [-4.2%, 6.2%]	1.1% [0.3%, 1.9%]

© 2023 Chua KP et al. *JAMA Health Forum.*

eFigure 1. Sample inclusion and exclusion criteria

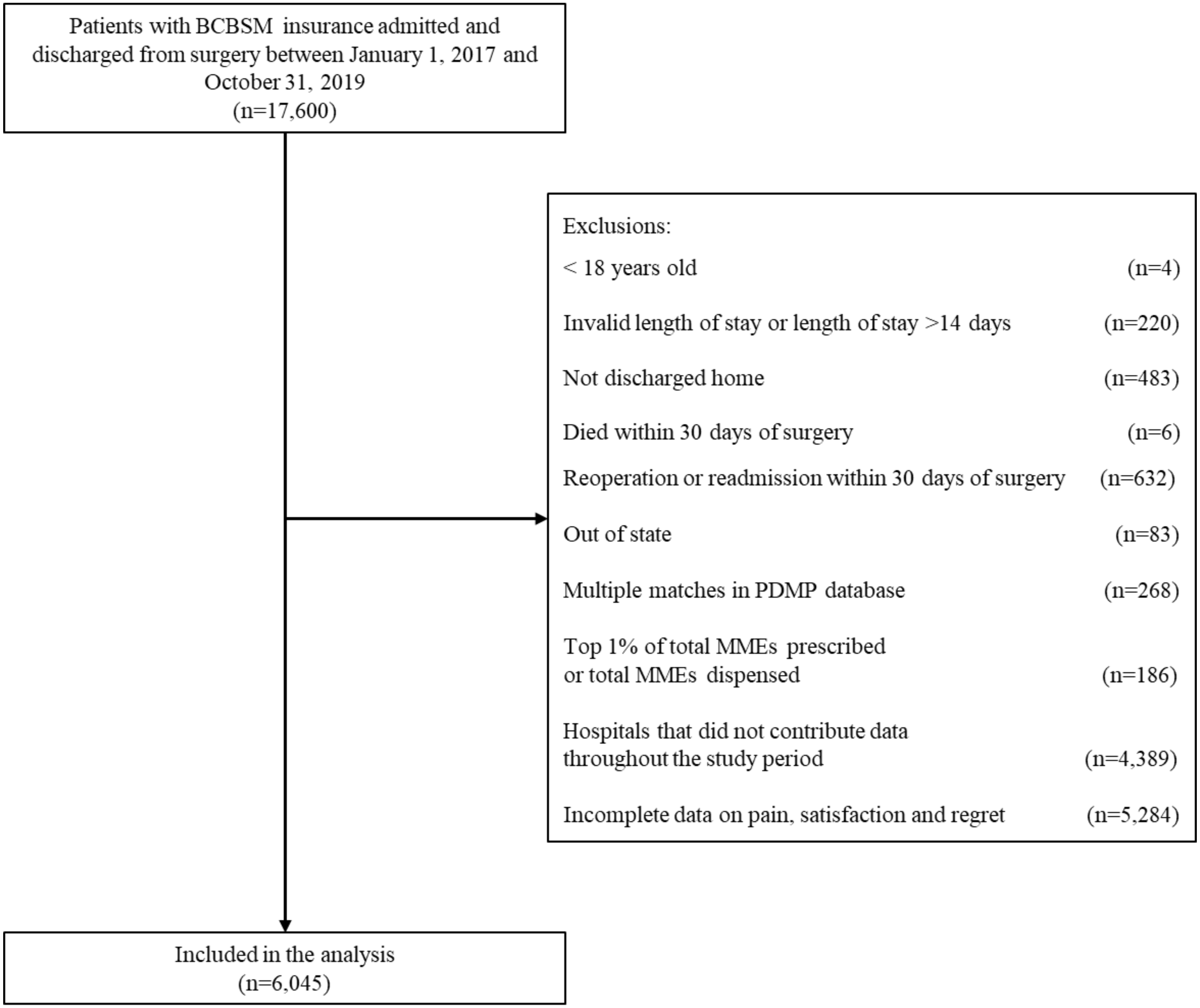

© 2023 Chua KP et al. *JAMA Health Forum.*

eFigure 2. Graphs for all other outcomes

Monthly proportion of patients with a discharge opioid prescription

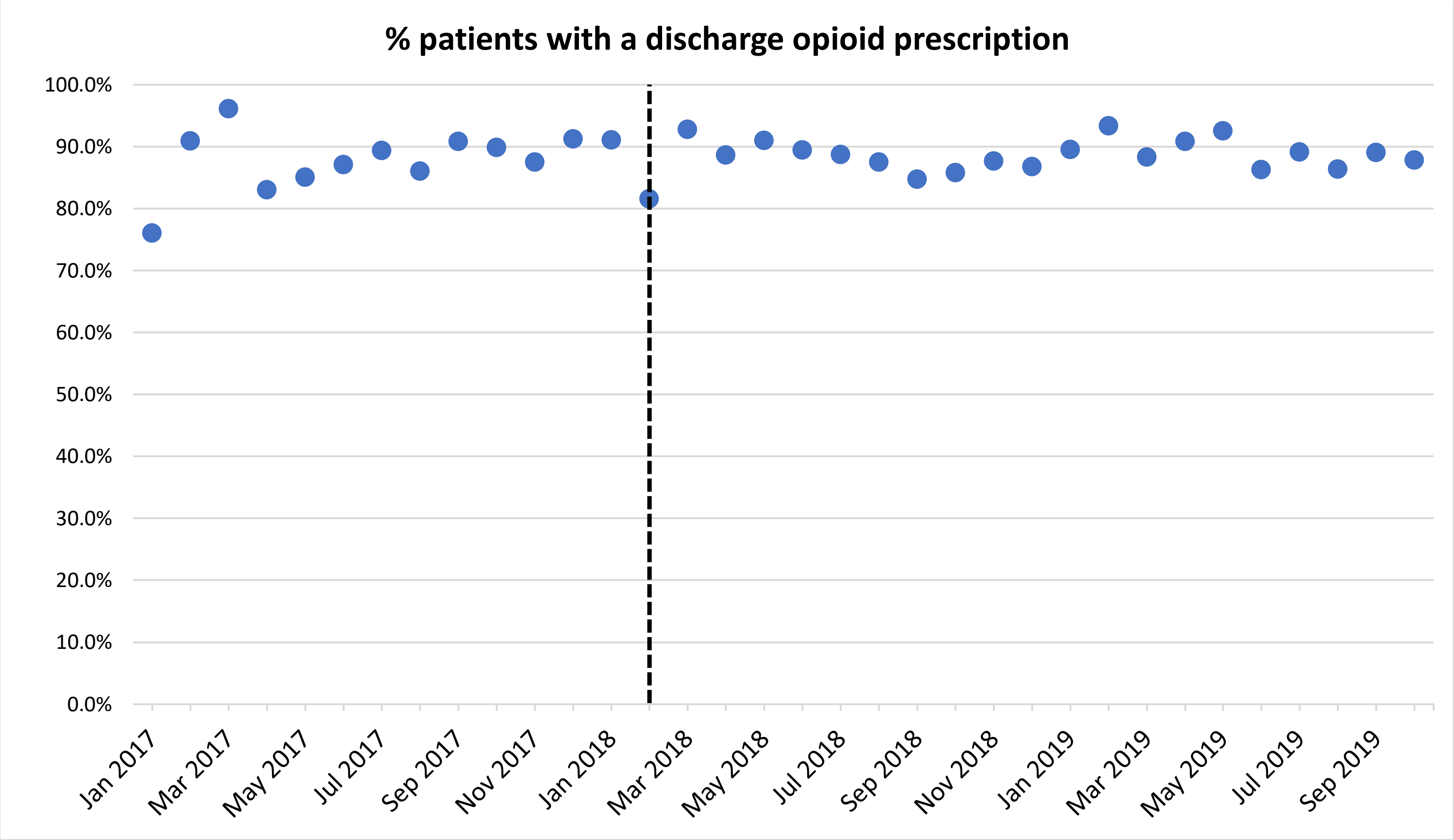

© 2023 Chua KP et al. *JAMA Health Forum.*

Monthly proportion of patients with a dispensed opioid prescription

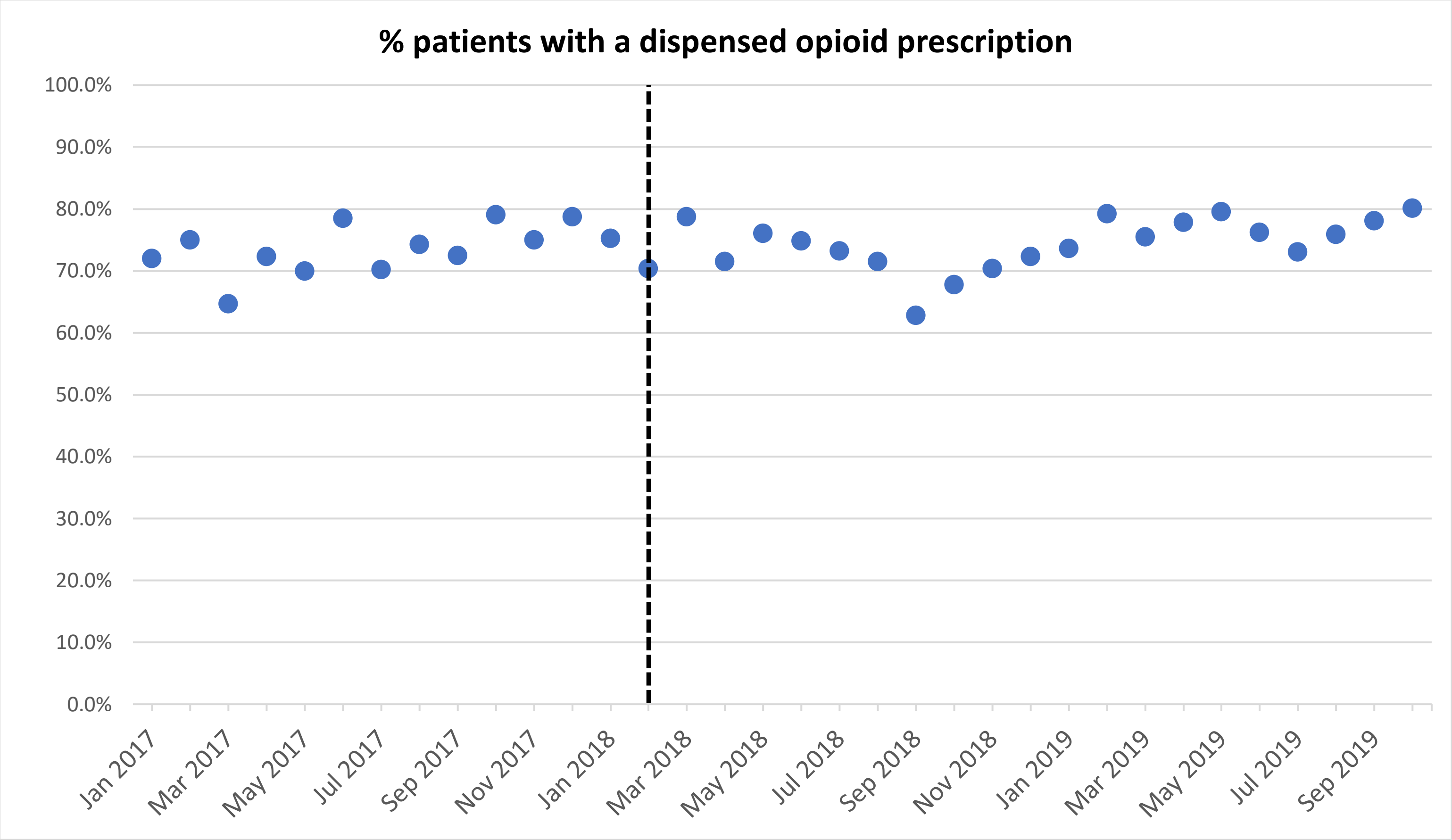

© 2023 Chua KP et al. *JAMA Health Forum.*

Monthly mean satisfaction with care

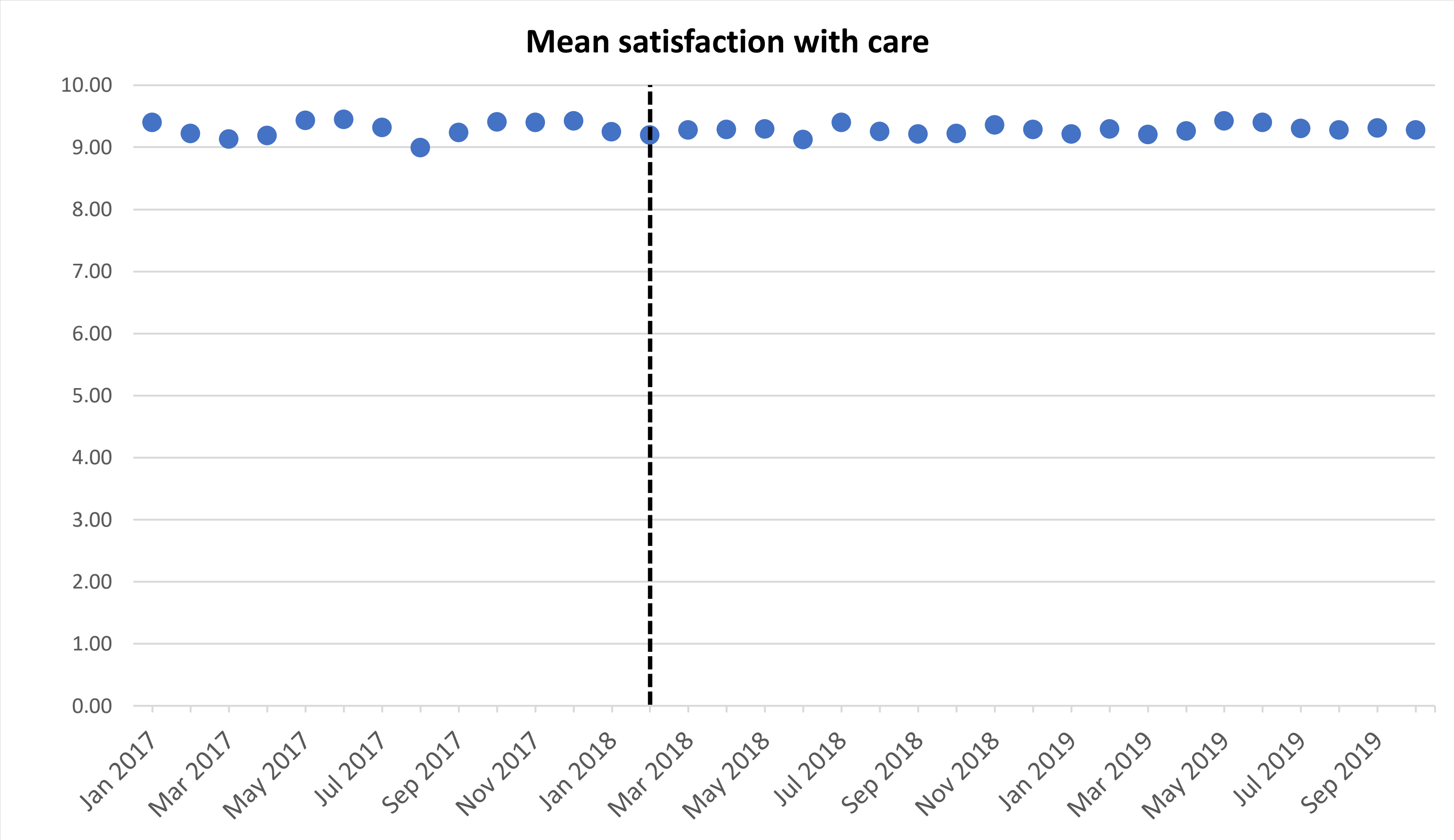

© 2023 Chua KP et al. *JAMA Health Forum.*

Monthly mean amount of regret regarding undergoing surgery

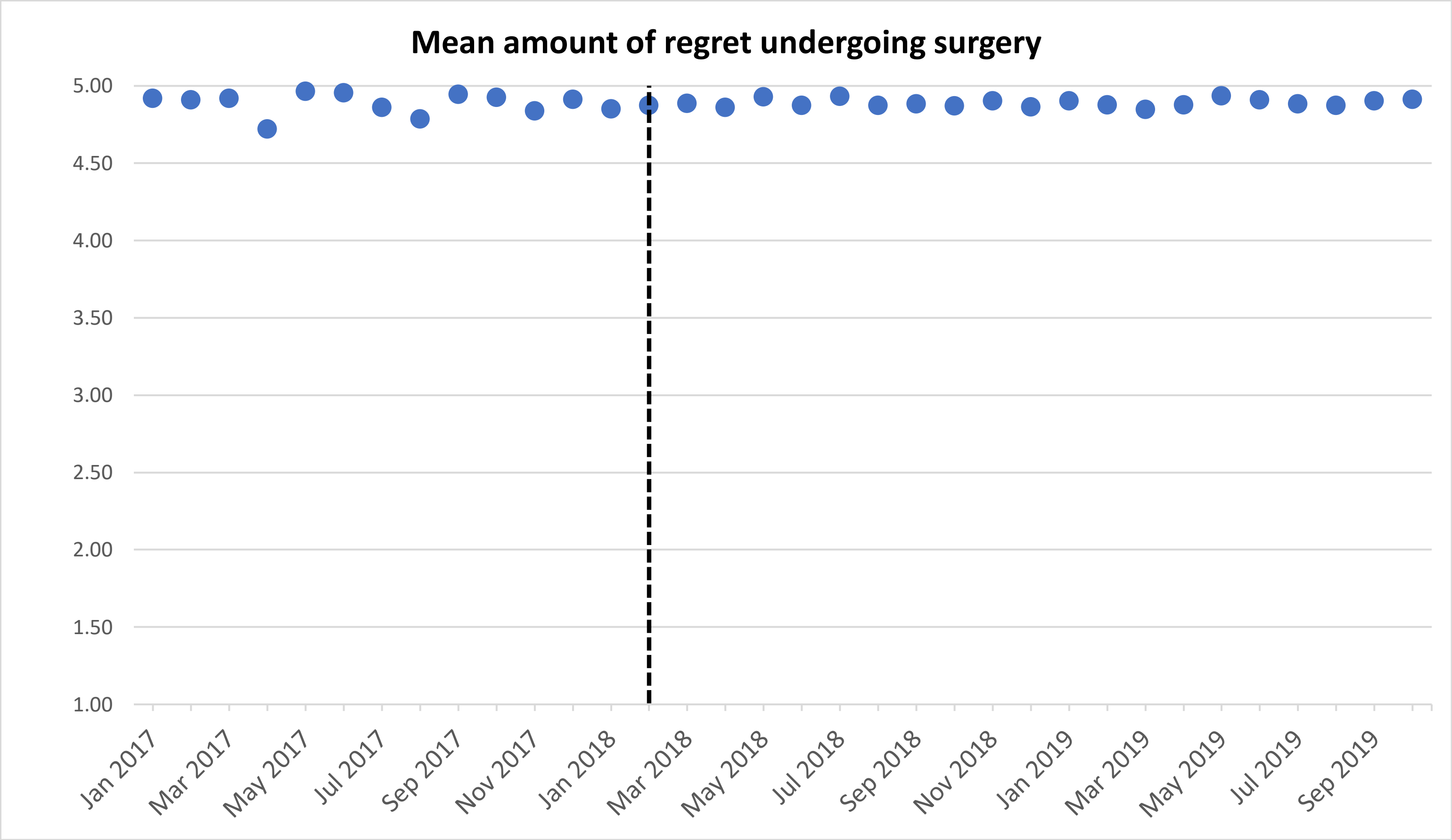

© 2023 Chua KP et al. *JAMA Health Forum.*

Monthly mean total morphine milligram equivalents in discharge opioid prescriptions

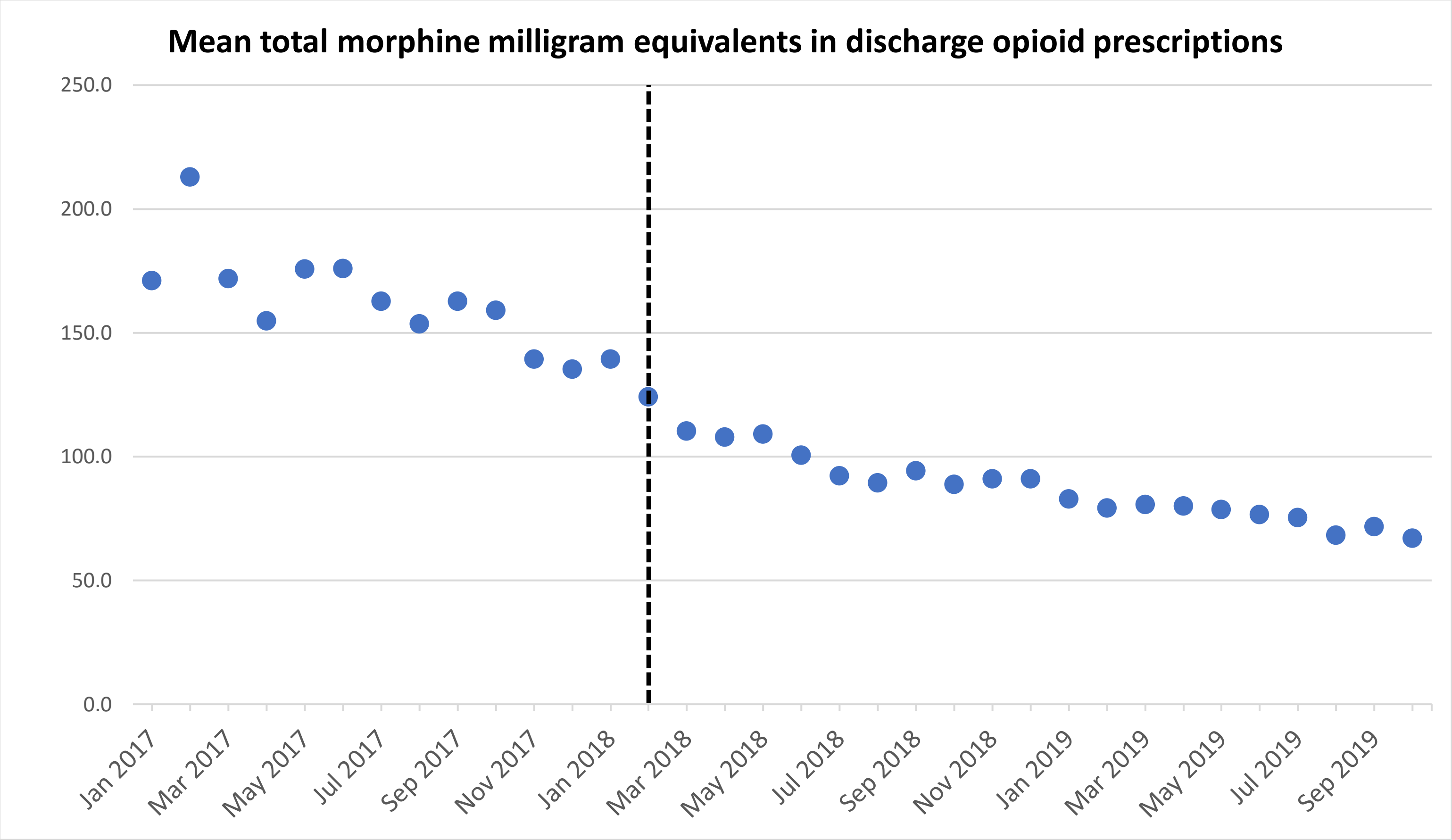

© 2023 Chua KP et al. *JAMA Health Forum.*

Monthly mean days supplied in discharge opioid prescriptions

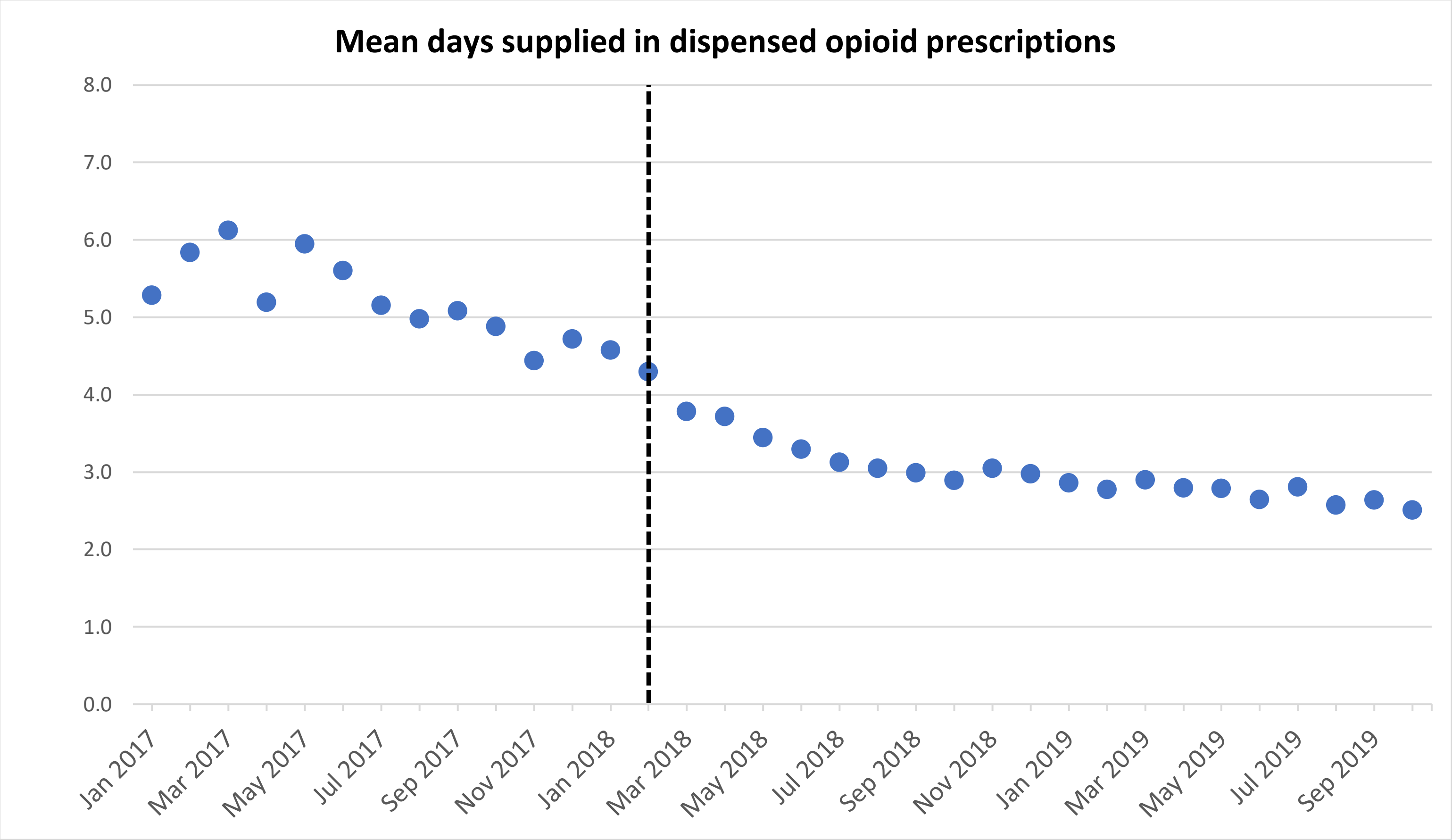

© 2023 Chua KP et al. *JAMA Health Forum.*

Monthly proportion of patients with at least one refill

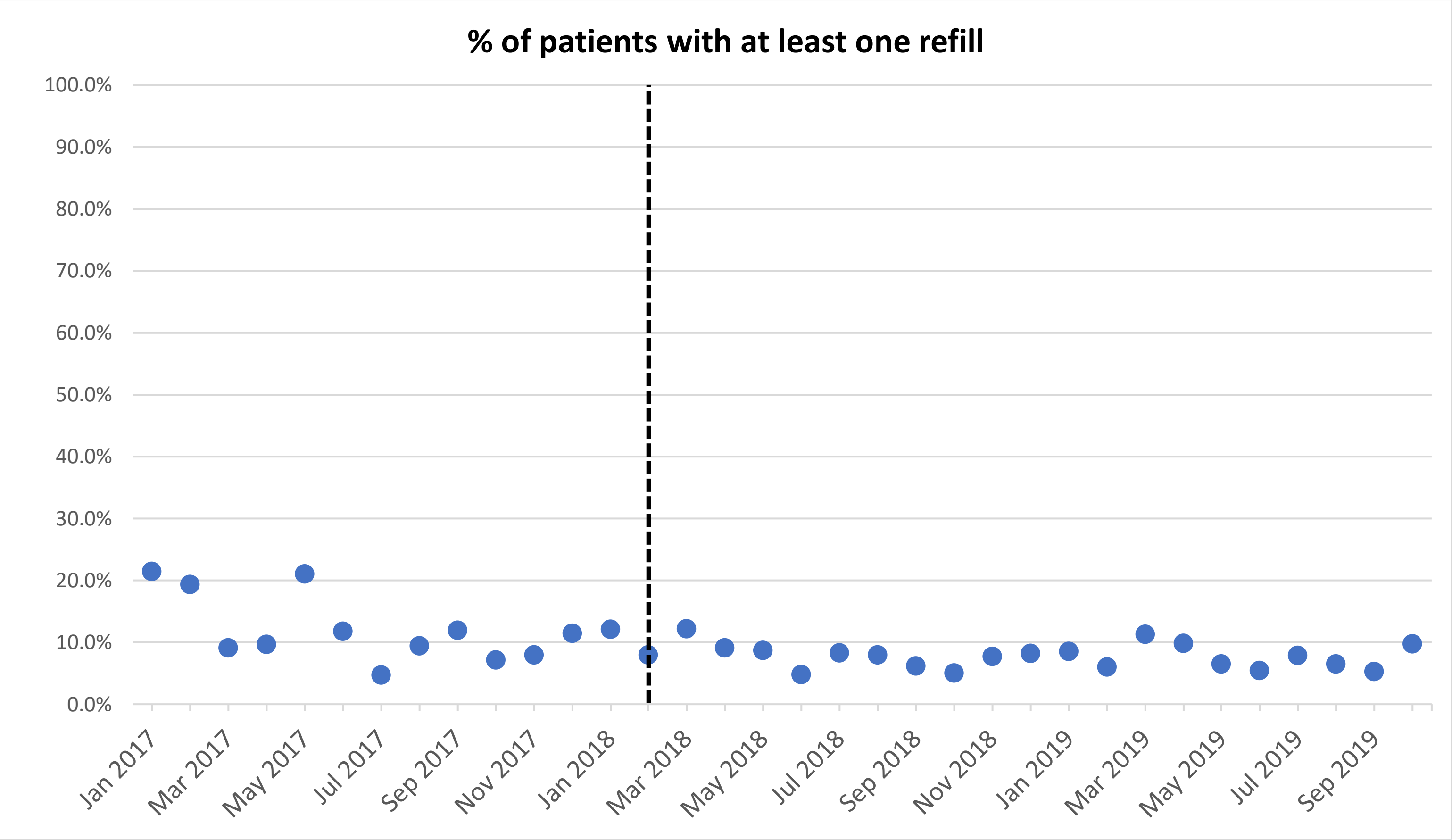

© 2023 Chua KP et al. *JAMA Health Forum.*

eFigure 3. Graphs for all ten outcomes with fitted lines

Mean patient-reported pain the first week after surgery

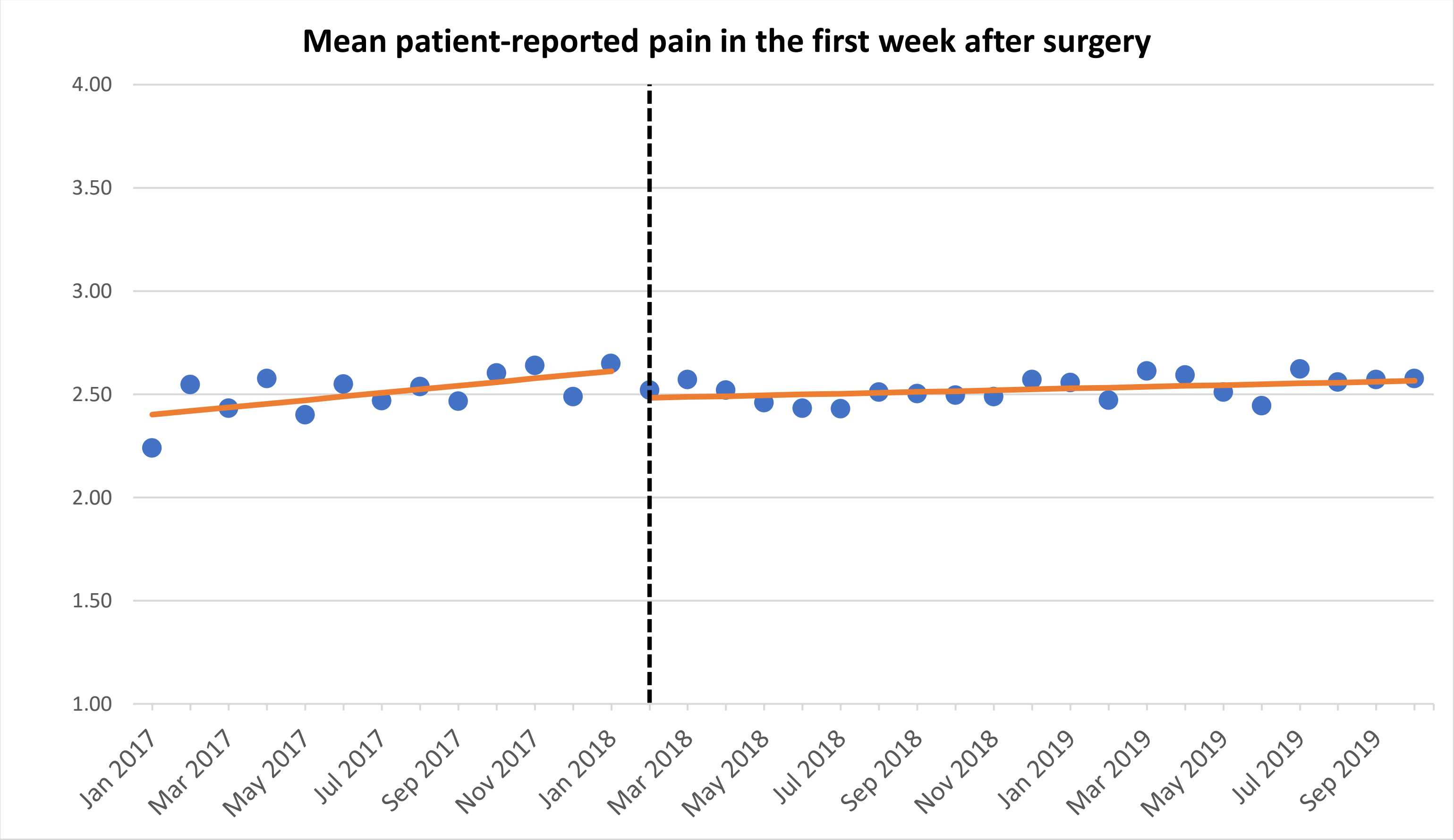

© 2023 Chua KP et al. *JAMA Health Forum.*

Mean total MMEs in dispensed opioid prescriptions

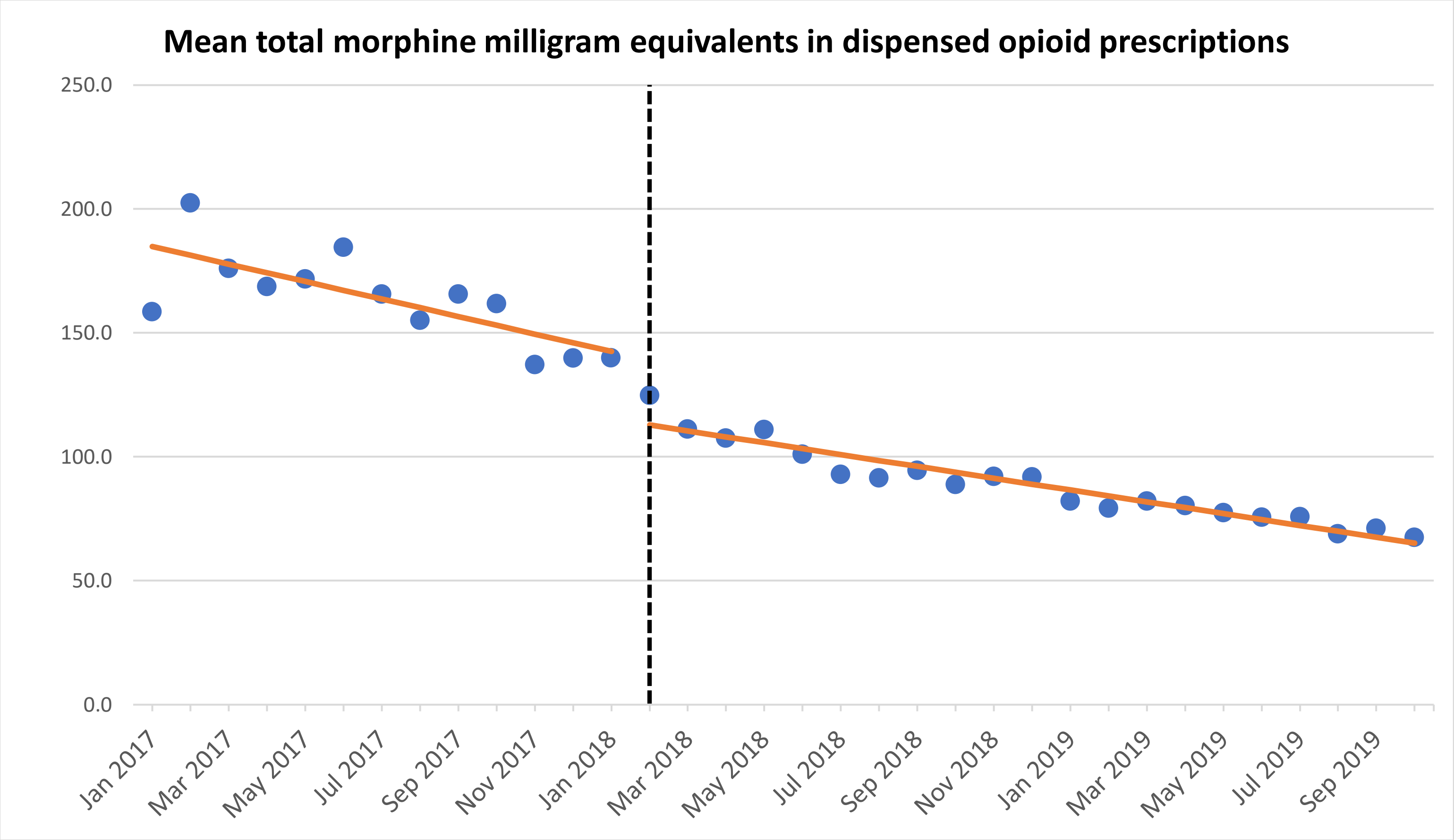

© 2023 Chua KP et al. *JAMA Health Forum.*

Proportion of patients with a dispensed opioid prescription exceeding a 5-day supply

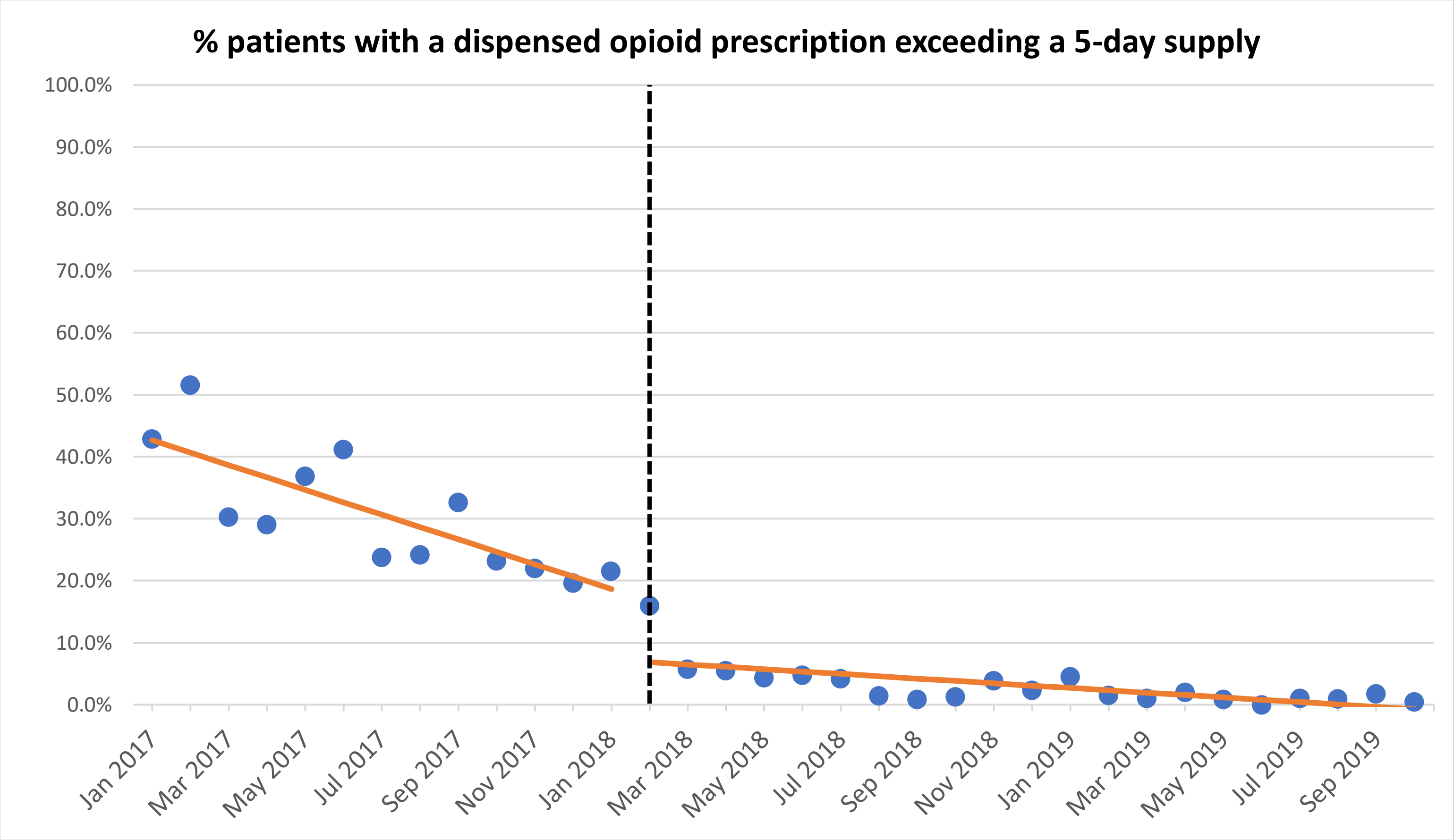

© 2023 Chua KP et al. *JAMA Health Forum.*

Monthly proportion of patients with a discharge opioid prescription

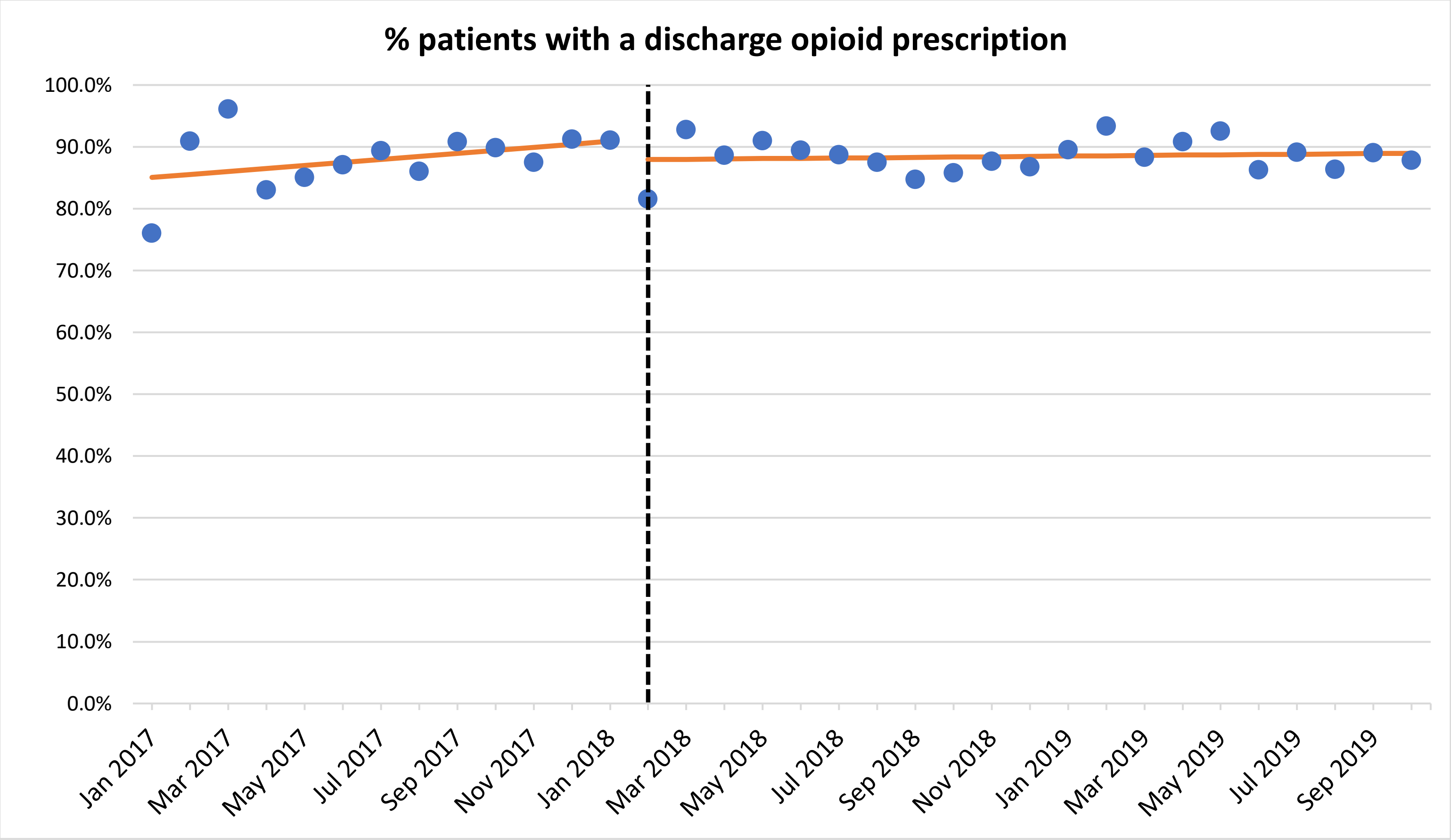

© 2023 Chua KP et al. *JAMA Health Forum.*

Monthly proportion of patients with a dispensed opioid prescription

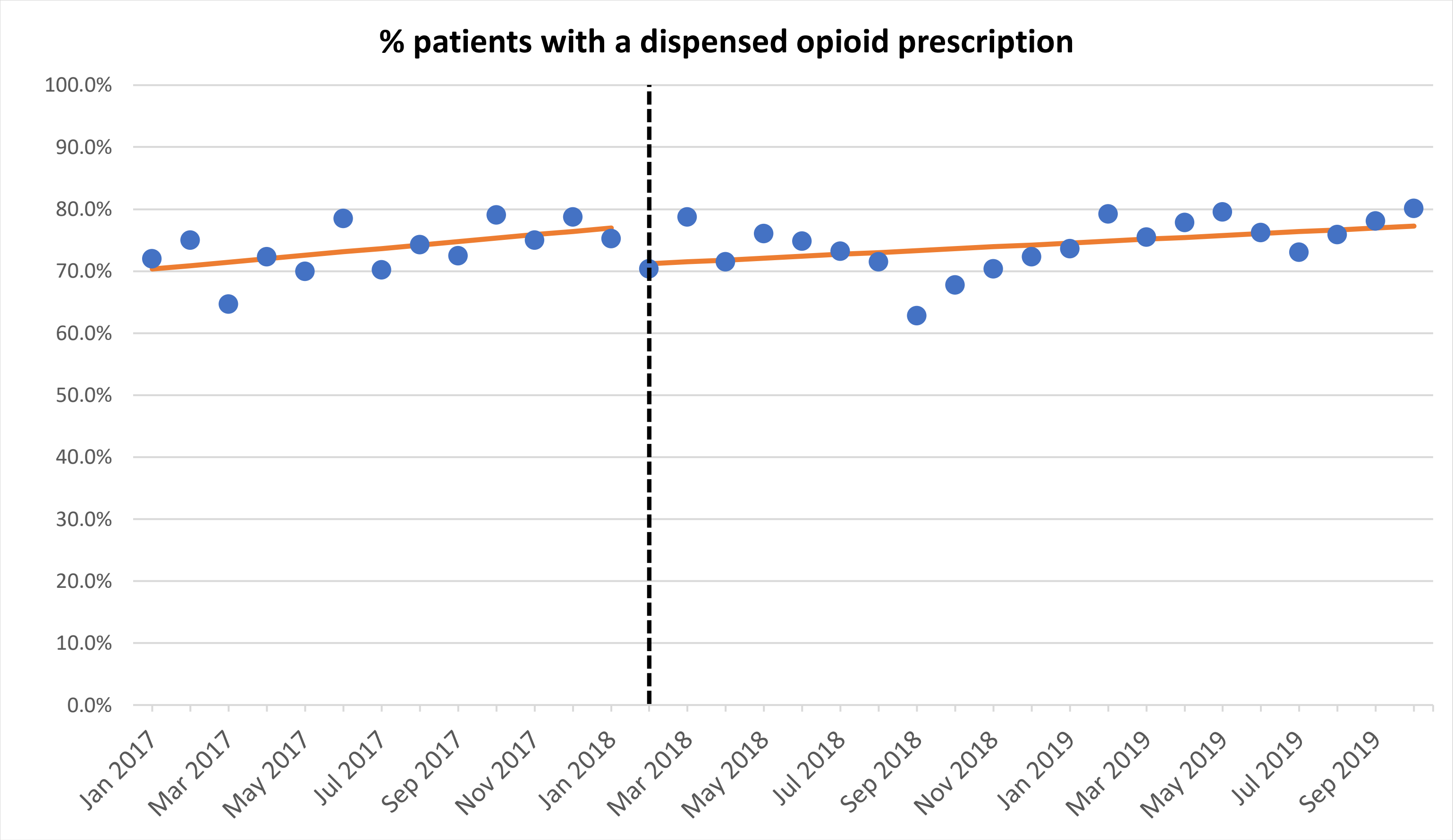

© 2023 Chua KP et al. *JAMA Health Forum.*

Monthly mean satisfaction with care

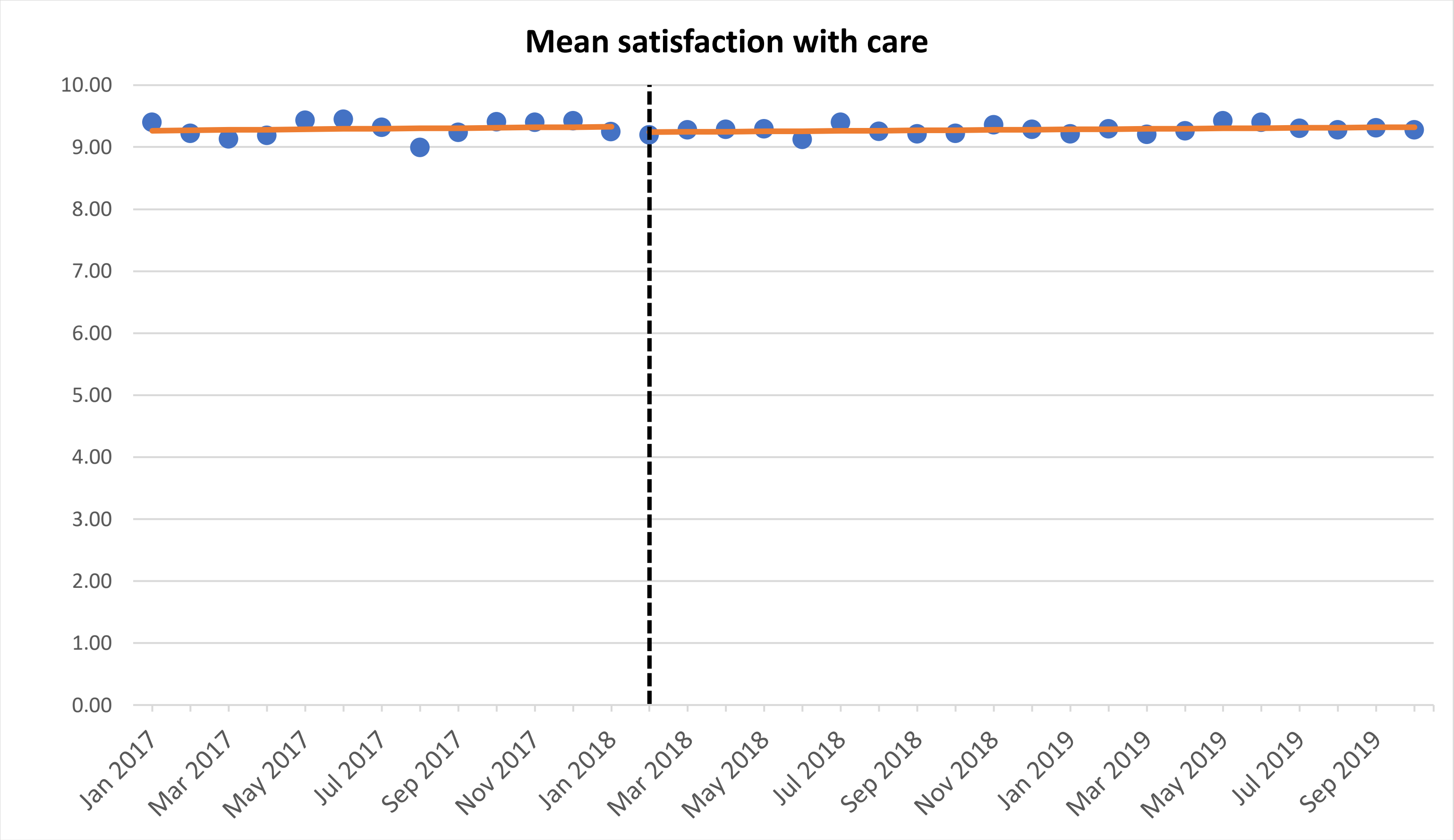

© 2023 Chua KP et al. *JAMA Health Forum.*

Monthly mean amount of regret regarding undergoing surgery

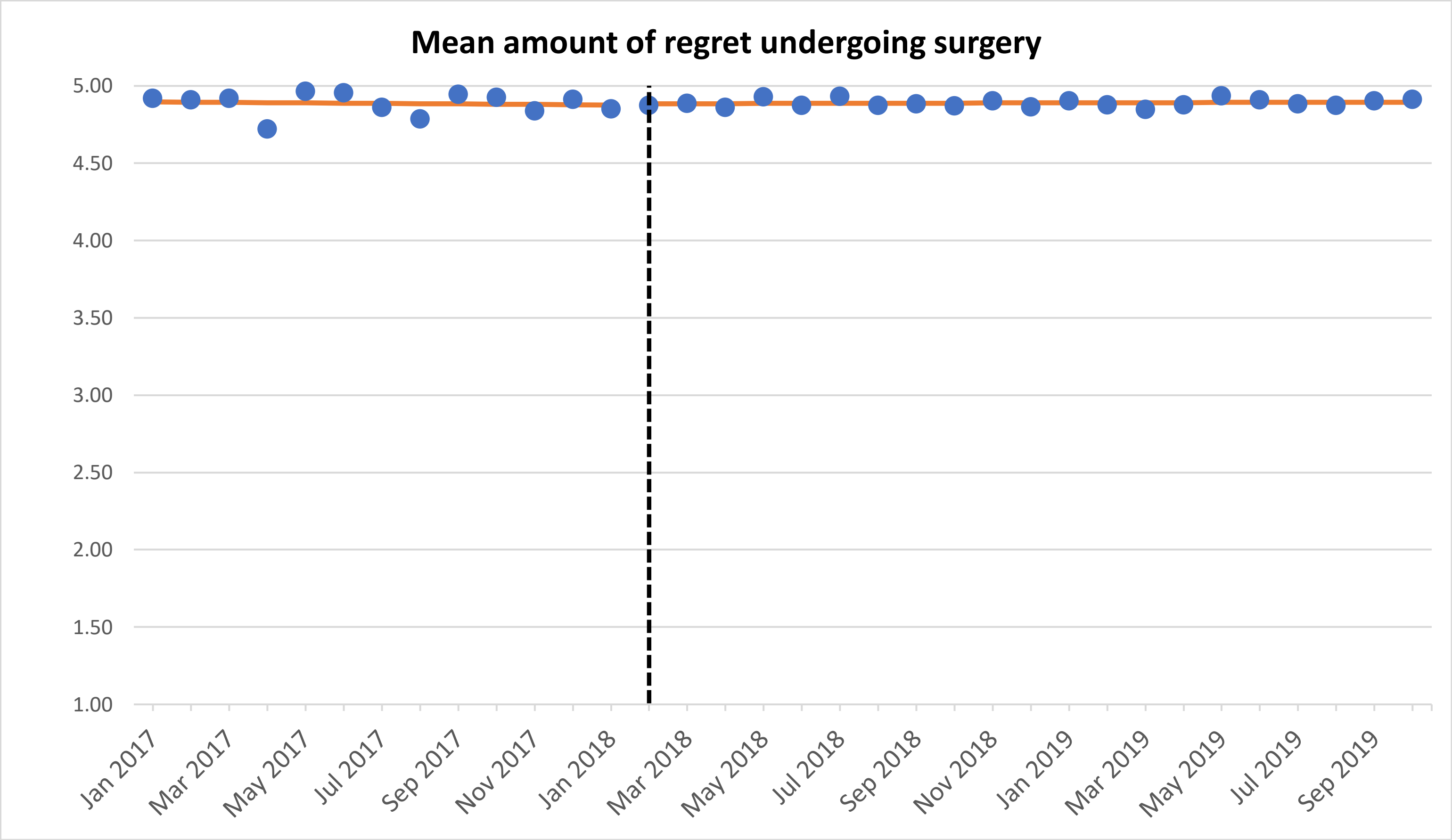

© 2023 Chua KP et al. *JAMA Health Forum.*

Monthly mean total morphine milligram equivalents in discharge opioid prescriptions

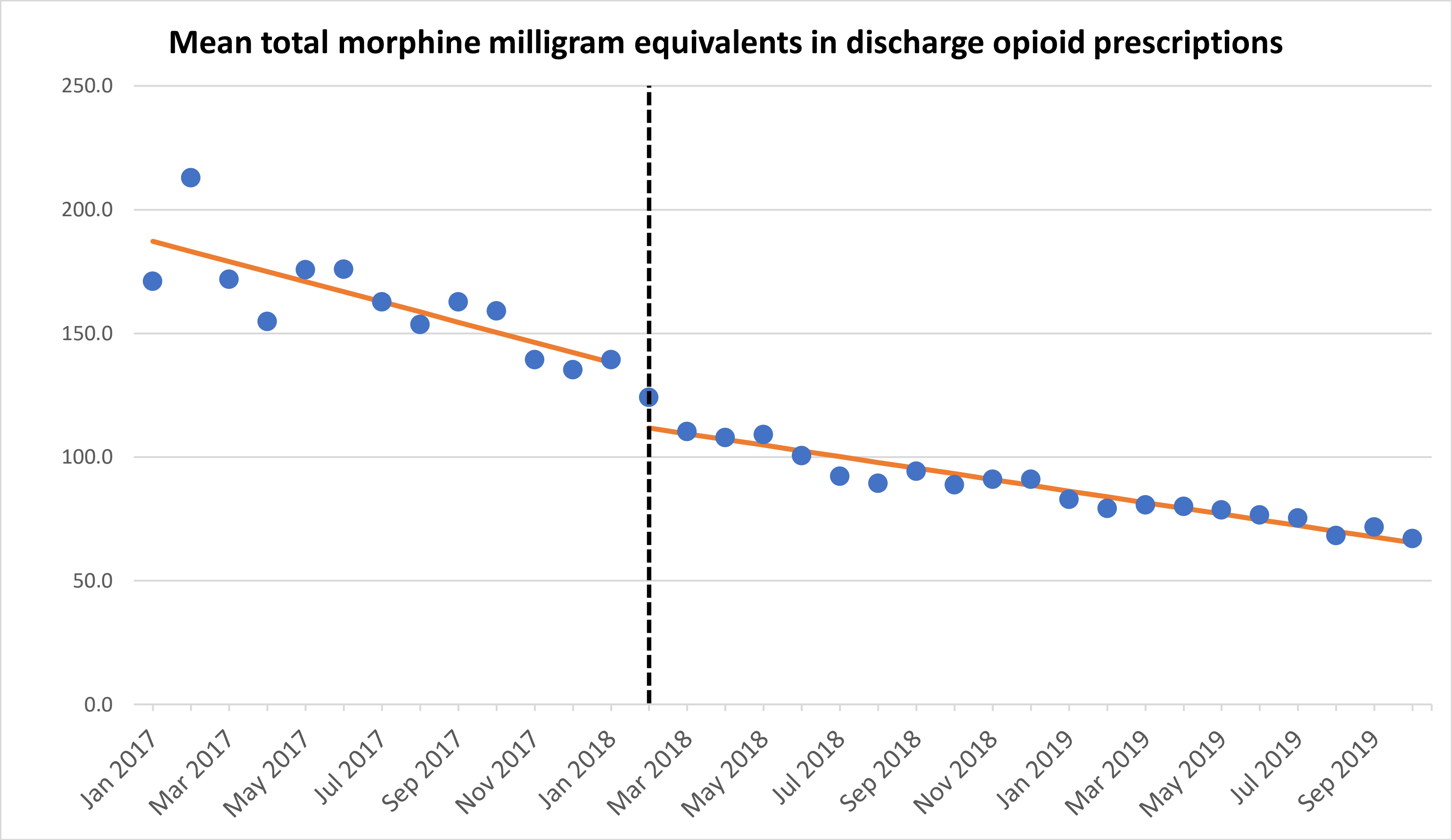

© 2023 Chua KP et al. *JAMA Health Forum.*

Monthly mean days supplied in discharge opioid prescriptions

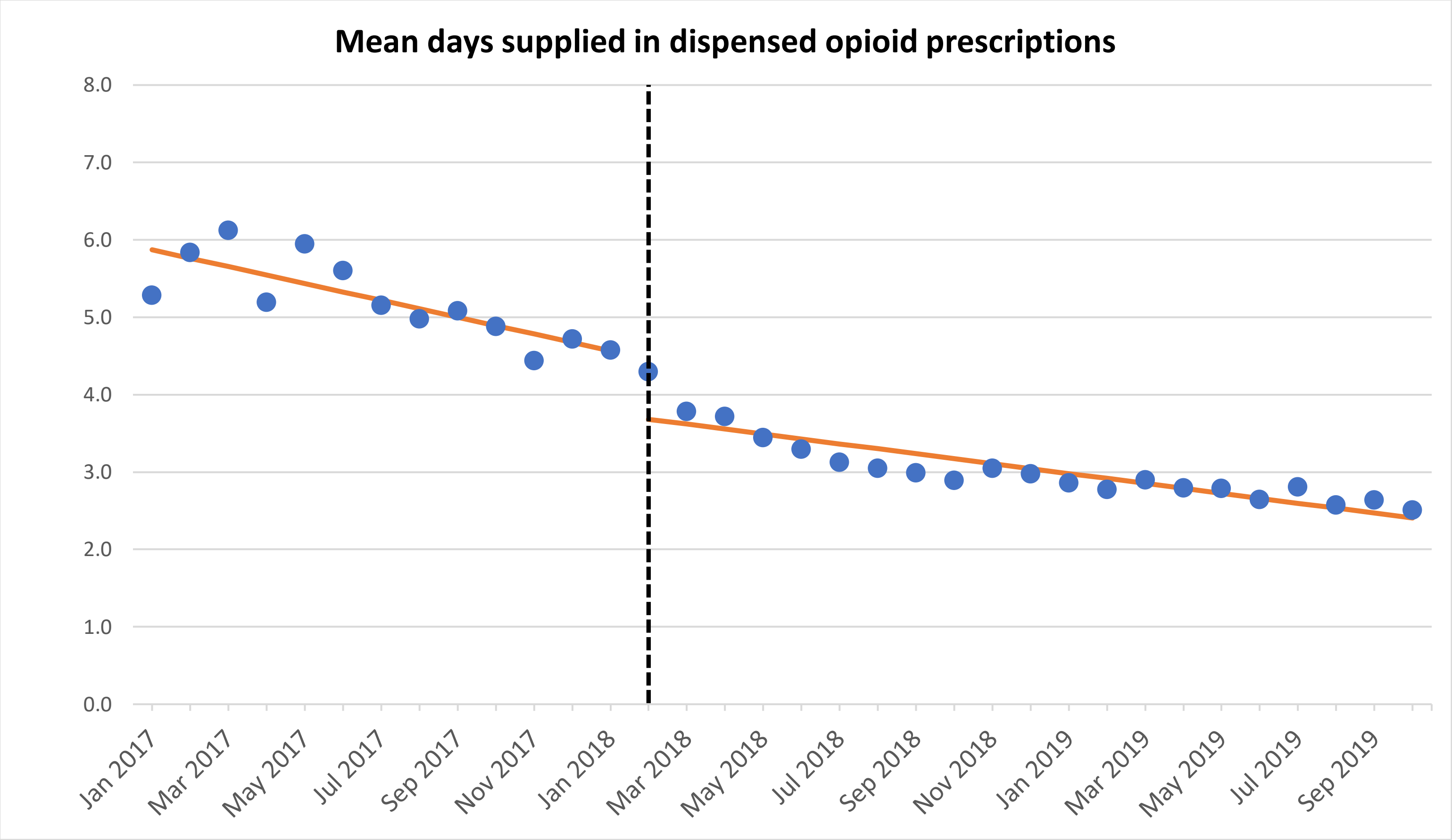

© 2023 Chua KP et al. *JAMA Health Forum.*

Monthly proportion of patients with at least one refill

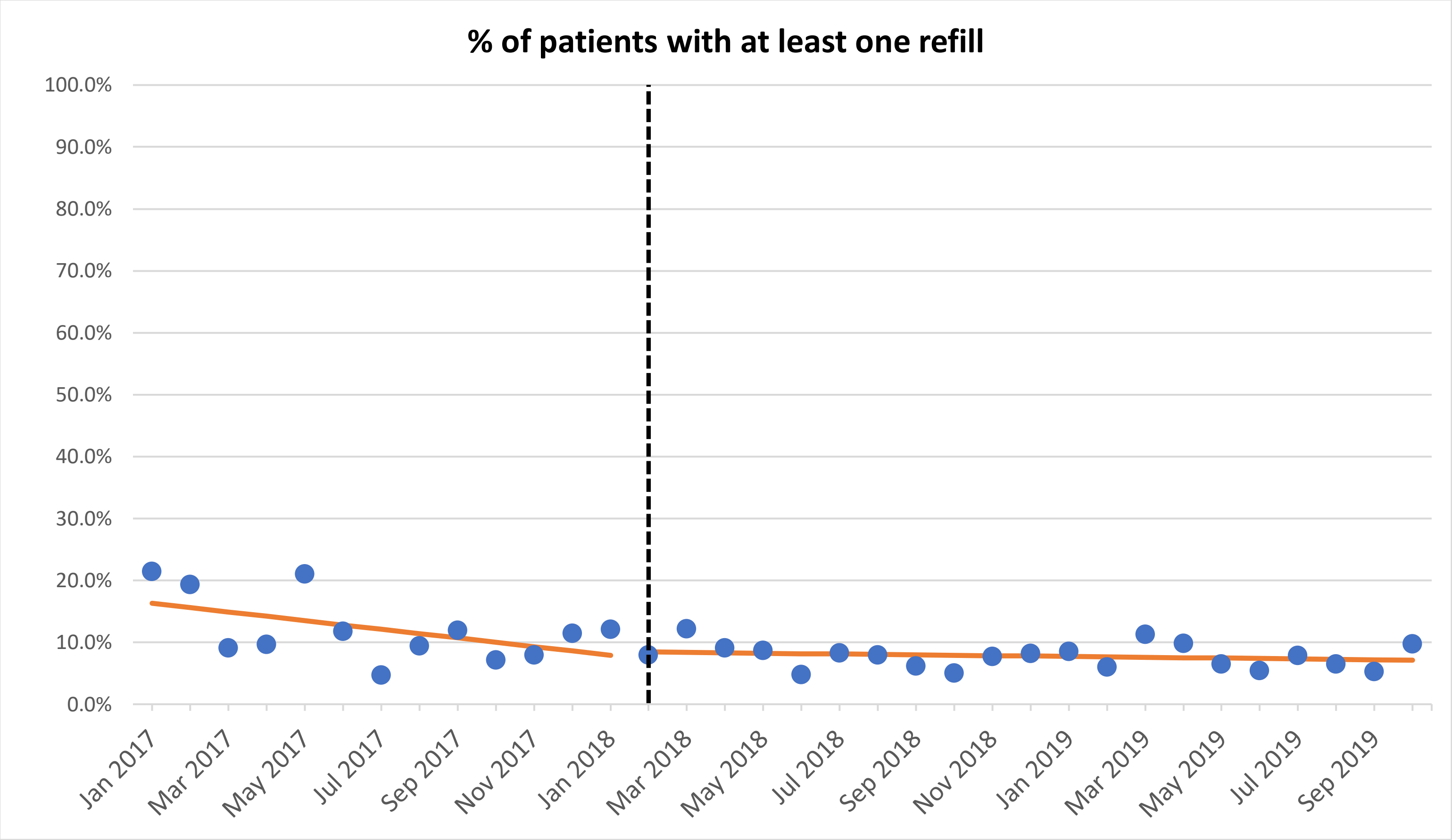

© 2023 Chua KP et al. *JAMA Health Forum.*

eFigure 4. Graphs of outcomes for opioid-naïve patients

Mean total MMEs in dispensed opioid prescriptions

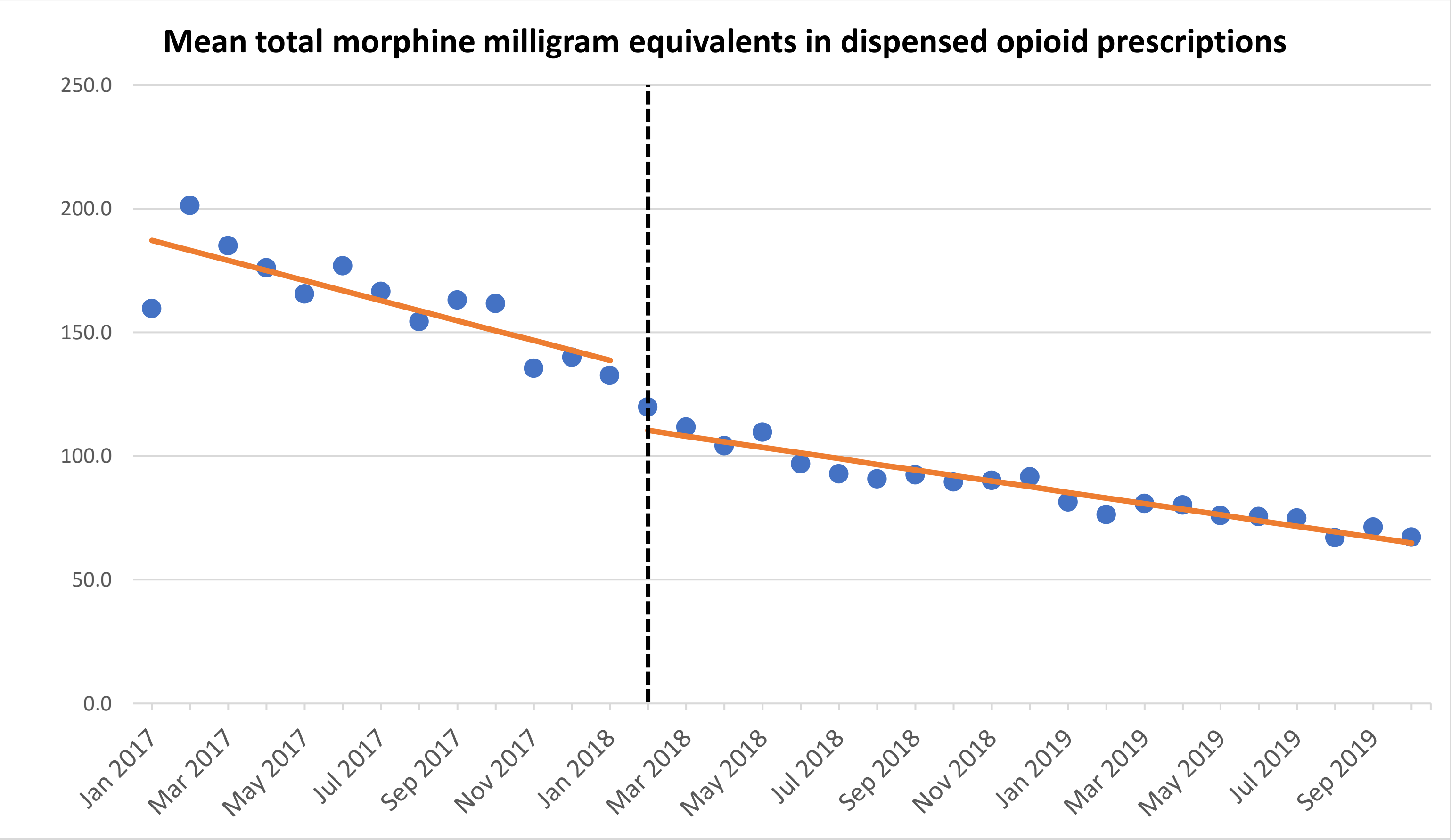

© 2023 Chua KP et al. *JAMA Health Forum.*

Mean patient-reported pain the first week after surgery

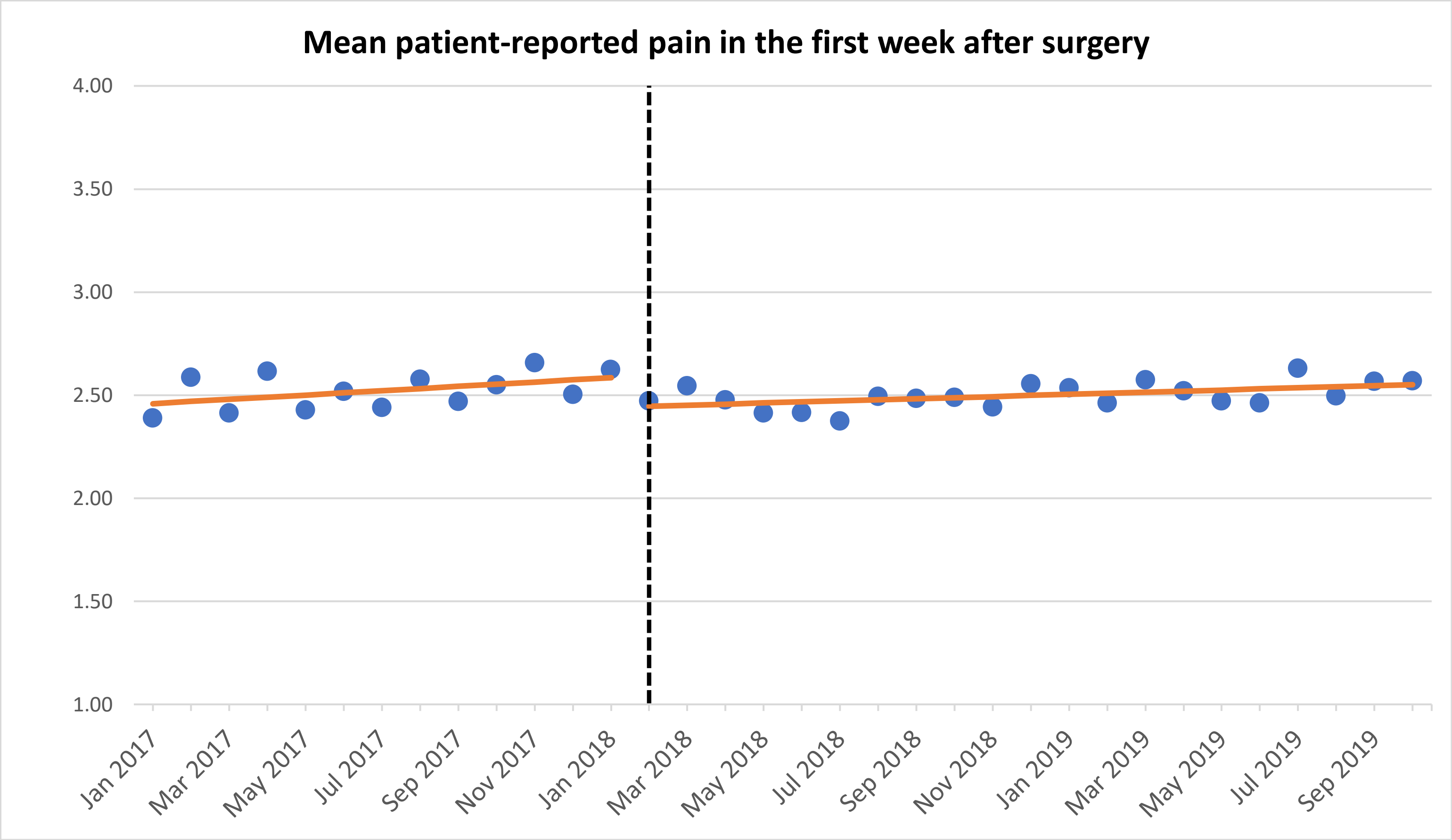

© 2023 Chua KP et al. *JAMA Health Forum.*

Proportion of patients with a dispensed opioid prescription exceeding a 5-day supply

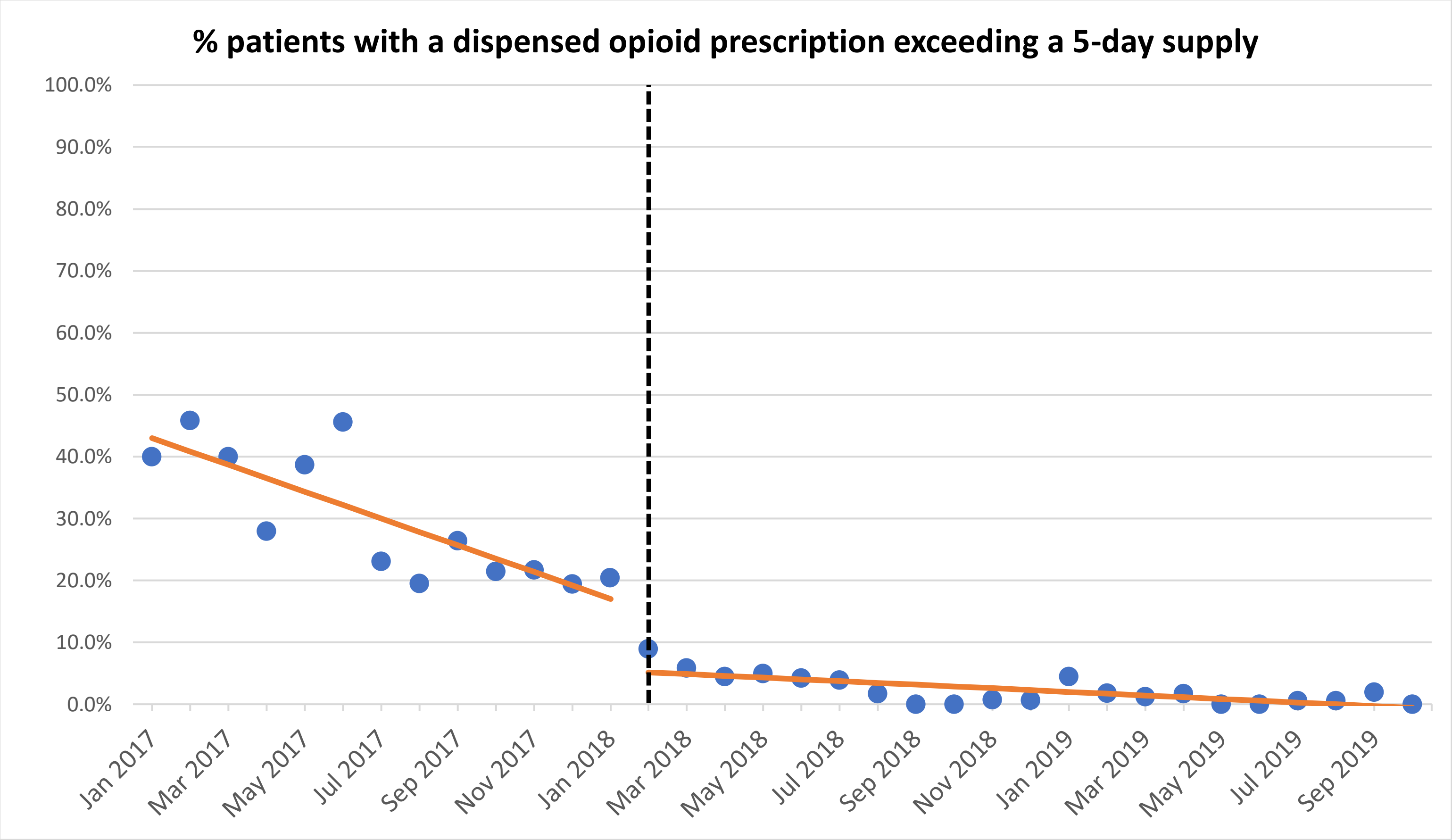

© 2023 Chua KP et al. *JAMA Health Forum.*

Monthly proportion of patients with a discharge opioid prescription

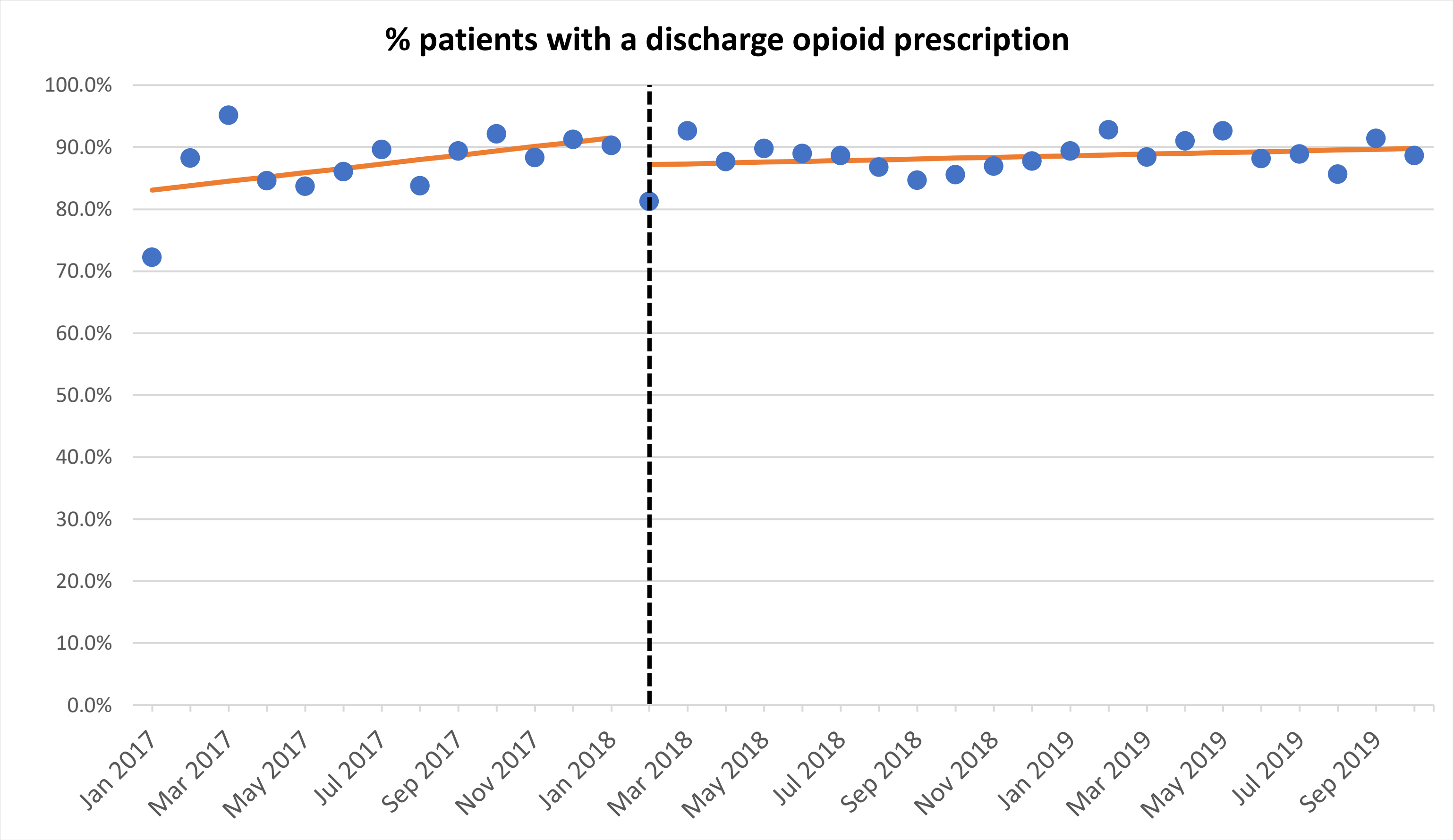

© 2023 Chua KP et al. *JAMA Health Forum.*

Monthly proportion of patients with a dispensed opioid prescription

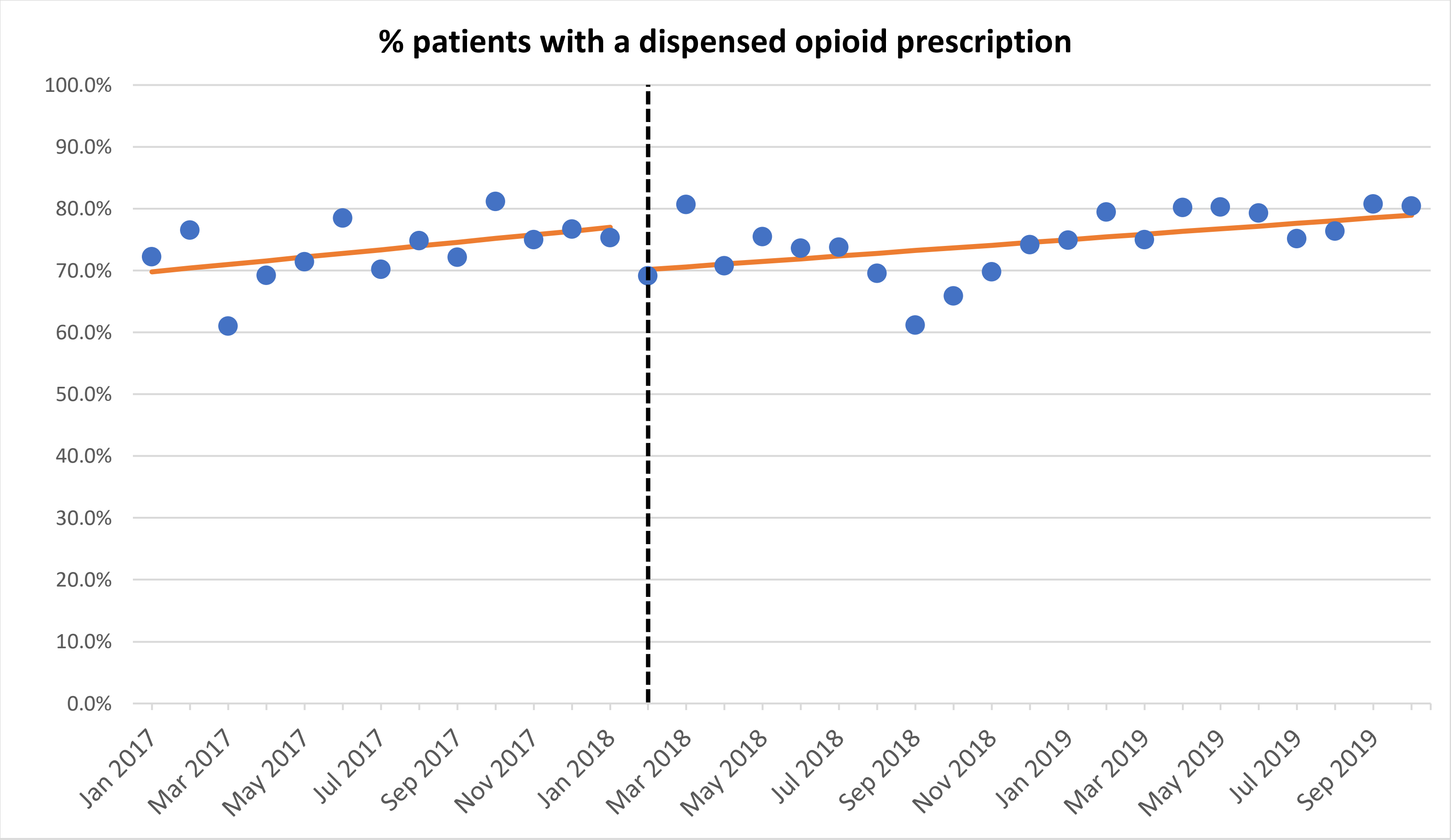

© 2023 Chua KP et al. *JAMA Health Forum.*

Monthly mean satisfaction with care

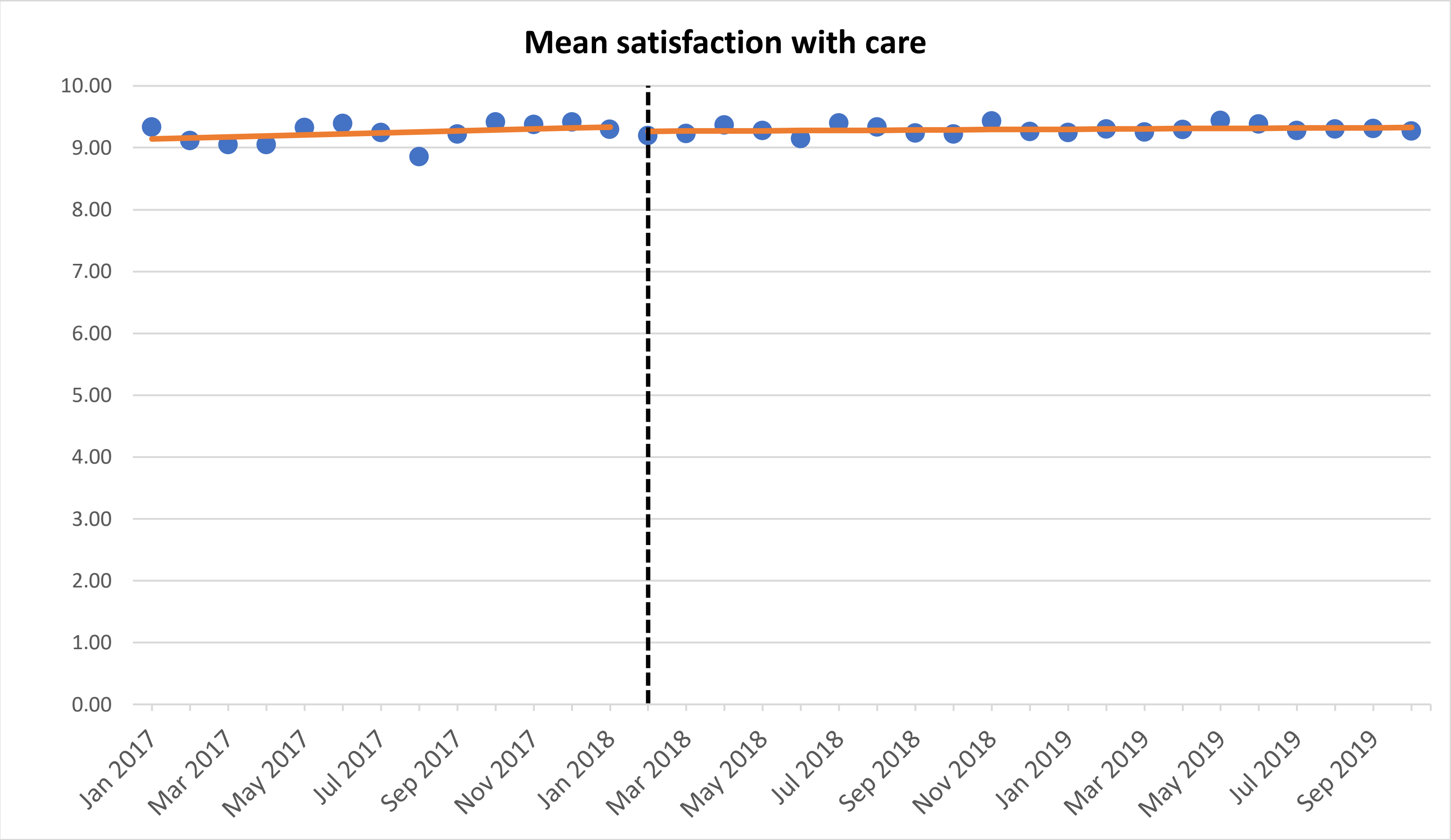

© 2023 Chua KP et al. *JAMA Health Forum.*

Monthly mean amount of regret regarding undergoing surgery

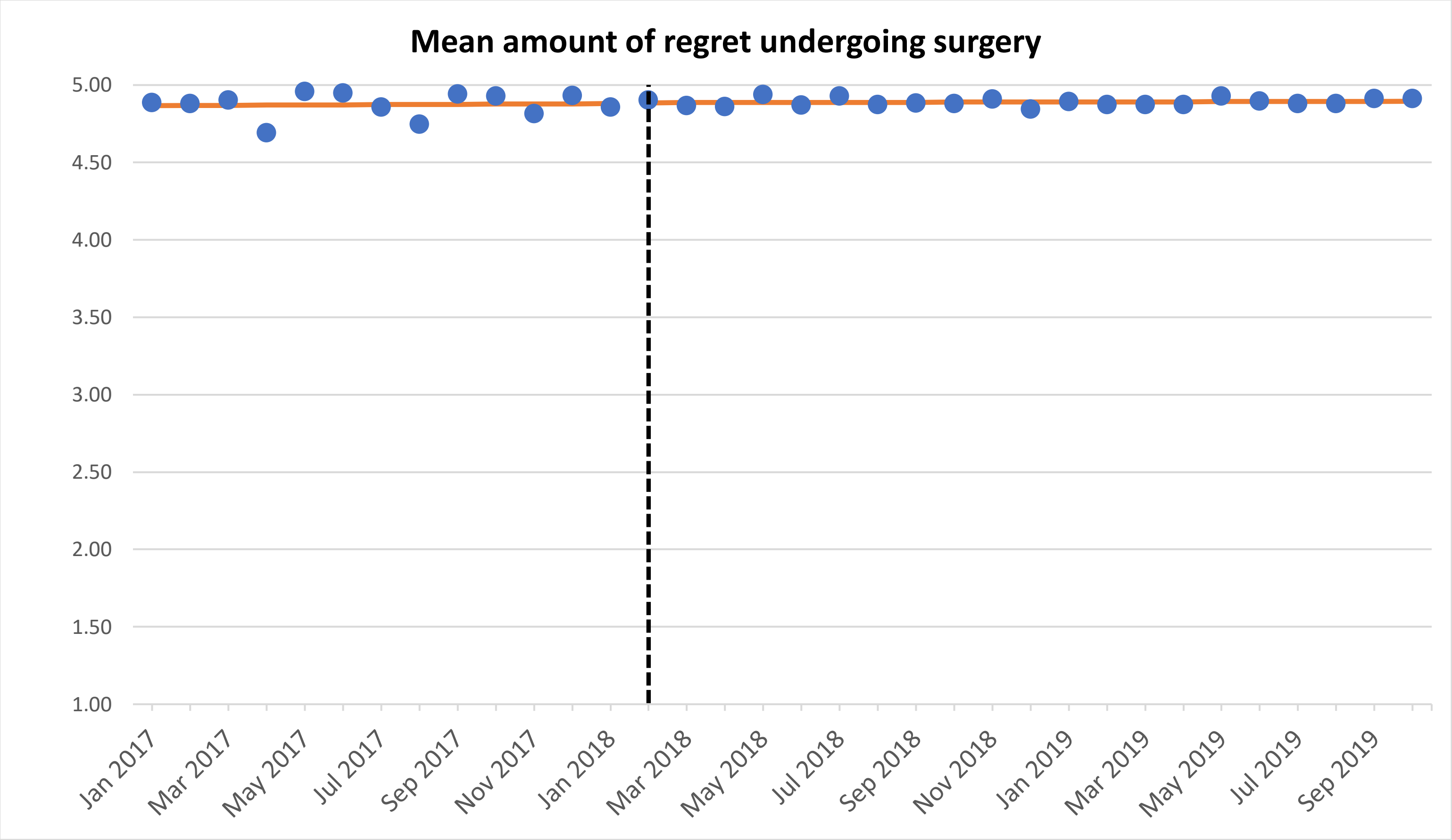

© 2023 Chua KP et al. *JAMA Health Forum.*

Monthly mean total morphine milligram equivalents in discharge opioid prescriptions

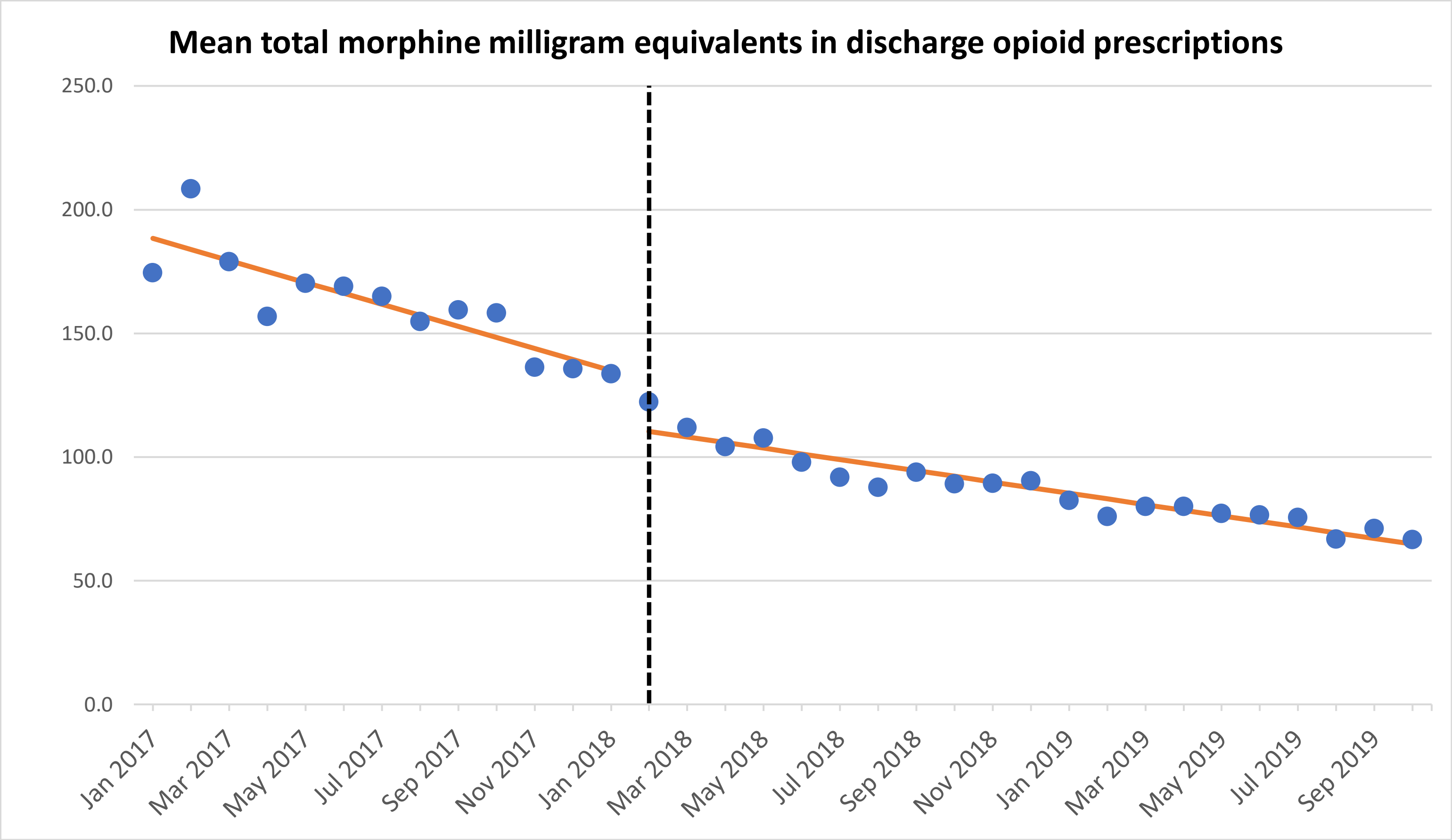

© 2023 Chua KP et al. *JAMA Health Forum.*

Monthly mean days supplied in discharge opioid prescriptions

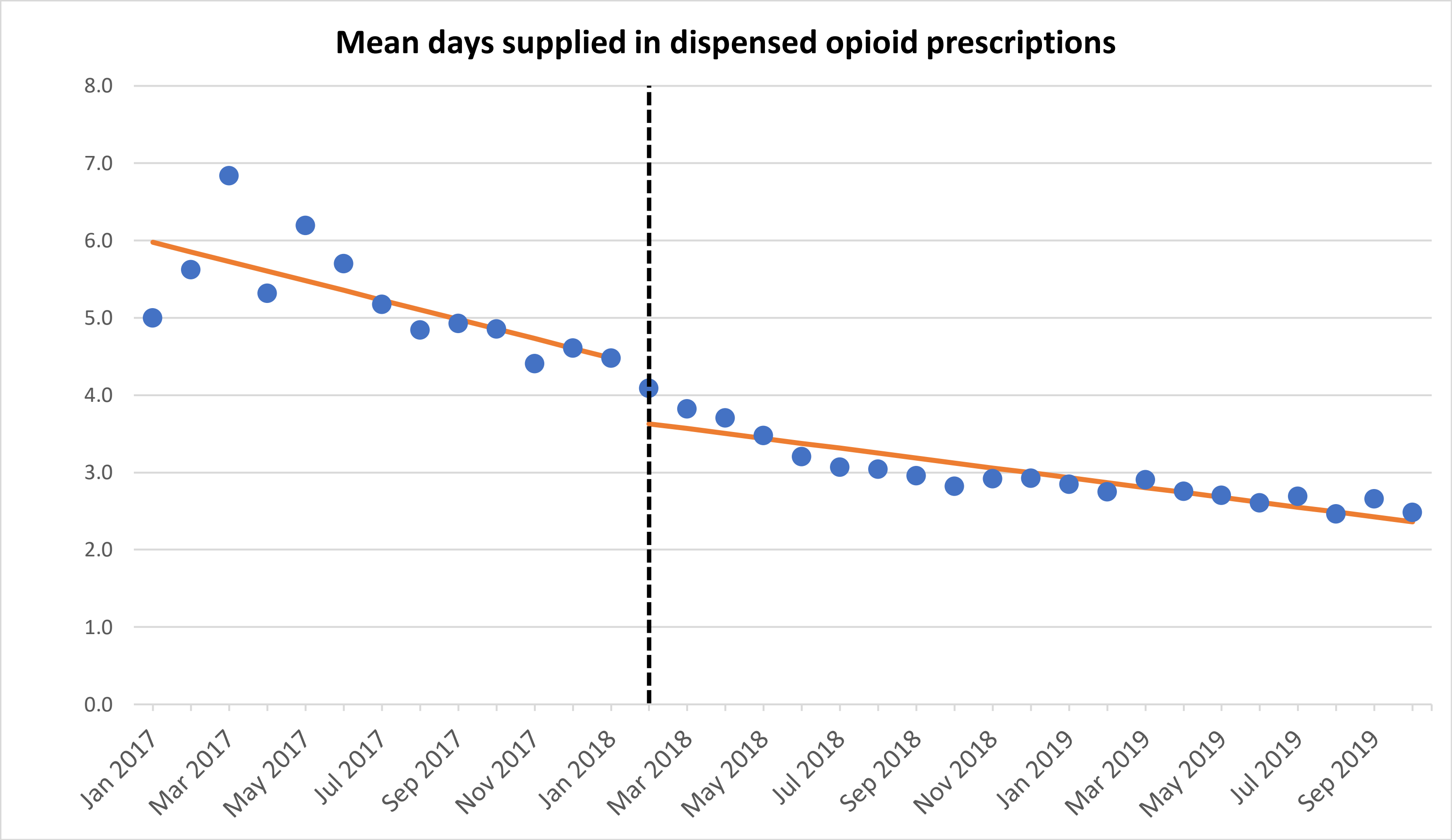

© 2023 Chua KP et al. *JAMA Health Forum.*

Monthly proportion of patients with at least one refill

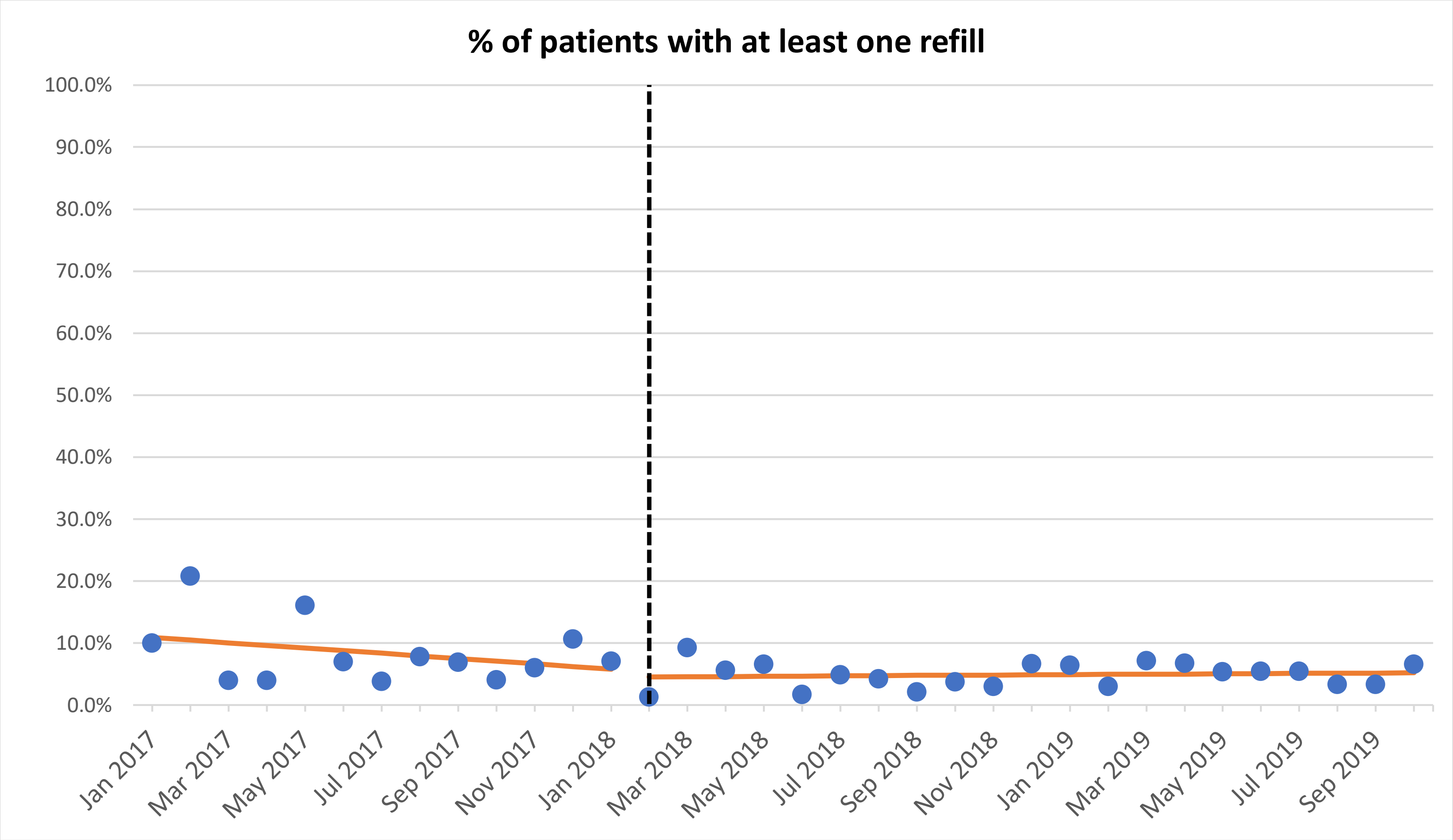

© 2023 Chua KP et al. *JAMA Health Forum.*

eTable 1. Subgroup analyses for opioid-naïve patients (n = 5,111)

Outcome	Intercept [95% CI]	Pre-intervention slope [95% CI]	Level change in February 2018 [95%CI]	Slope change in February 2018 [95% CI]
% patients with a discharge opioid prescription[a]	83.1% [74.6%, 91.5%]	0.7% [-0.3%, 1.7%]	-5.0% [-10.7%, 0.7%]	-0.6% [-1.6%, 0.4%]
% patients with a dispensed opioid prescription[b]	69.8% [63.8%, 75.8%]	0.6% [-0.09%, 1.3%]	-7.4% [-13.5%, -1.3%]	-0.2% [-0.9%, 0.6%]
Mean pain in the first week of surgery (scale of 1-4)	2.5 [2.4, 2.5]	0.01 [0.004, 0.02]	-0.1 [-0.2, -0.07]	-0.005 [-0.01, 0.003]
Mean satisfaction with care (scale of 0-10)	9.1 [9, 9.3]	0.02 [-0.002, 0.03]	-0.08 [-0.2, 0.07]	-0.01 [-0.03, 0.006]
Mean amount of regret regarding undergoing surgery (scale of 1-5)	4.9 [4.8, 4.9]	0.001 [-0.005, 0.008]	0.004 [-0.05, 0.06]	-0.0006 [-0.007, 0.006]
Mean total morphine milligram equivalents in the discharge opioid prescription[c]	188.4 [179.4, 197.4]	-4.5 [-5.6, -3.3]	-20.1 [-30.6, -9.6]	2.2 [1, 3.4]
Mean total morphine milligram equivalents in the dispensed opioid prescription[c]	187.2 [167.6, 206.8]	-4.1 [-6.5, -1.6]	-24.2 [-38.8, -9.6]	1.8 [-0.7, 4.2]
% patients with dispensed opioid prescription > 5-day supply[c]	43.0% [39.8%, 46.1%]	-2.2% [-2.6%, -1.8%]	-9.7% [-13.5%, -5.9%]	1.9% [1.4%, 2.3%]
Mean days supplied in discharge opioid prescription[c]	6.0 [5.2, 6.8]	-0.1 [-0.2, -0.03]	-0.7 [-1.2, -0.2]	0.06 [-0.03, 0.2]
% patients with at least one refill[c]	10.9% [7.1%, 14.7%]	-0.4% [-1.0%, 0.1%]	-0.8% [-5.4%, 3.7%]	0.5% [-0.07%, 1.0%]

Opioid-naïve patients were those without any dispensed opioid prescriptions in the prior 120 days.

[a]Defined as an opioid prescription prescribed at discharge from surgery

[b]Defined as an opioid prescription dispensed within 3 days of discharge from surgery (or within 3 days of discharge from hospitalization after surgery, as applicable)

[c]These 5 outcomes were only calculated for patients with both a discharge and dispensed opioid prescription

© 2023 Chua KP et al. *JAMA Health Forum.*

eTable 2. Subgroup analyses for non-opioid naïve patients (n = 934)

Outcome	Intercept [95% CI]	Pre-intervention slope [95% CI]	Level change in February 2018 [95%CI]	Slope change in February 2018 [95% CI]
% patients with a discharge opioid prescription[a]	91.3% [85.7%, 97.0%]	-0.2% [-1.1%, 0.7%]	4.2% [-6.0%, 14.3%]	-0.2% [-1.1%, 0.7%]
% patients with a dispensed opioid prescription[b]	73.1% [65.4%, 80.8%]	0.3% [-0.8%, 1.5%]	-0.09% [-11.2%, 11.0%]	-0.8% [-2.1%, 0.5%]
Mean pain in the first week of surgery (scale of 1-4)	2.2 [2.0, 2.4]	0.04 [0.01, 0.07]	-0.06 [-0.3, 0.1]	-0.04 [-0.07, -0.01]
Mean satisfaction with care (scale of 0-10)	9.8 [9.5, 10]	-0.04 [-0.07, -0.004]	-0.2 [-0.5, 0.1]	0.04 [0.008, 0.08]
Mean amount of regret regarding undergoing surgery (scale of 1-5)	5.0 [5.0, 5.0]	-0.01 [-0.02, -0.007]	0.03 [-0.03, 0.09]	0.01 [0.006, 0.02]
Mean total morphine milligram equivalents in the discharge opioid prescription[c]	185.2 [167.4, 202.9]	-2.8 [-4.8, -0.8]	-31.4 [-46.3, -16.4]	0.4 [-1.6, 2.5]
Mean total morphine milligram equivalents in the dispensed opioid prescription[c]	178.2 [157.2, 199.3]	-1.6 [-3.9, 0.7]	-33.7 [-52.3, -15.1]	-1.1 [-3.5, 1.3]
% patients with dispensed opioid prescription > 5-day supply[c]	40.5% [15.2%, 65.9%]	-1.2% [-4.0%, 1.7%]	-11.8% [-30.9%, 7.3%]	0.5% [-2.4%, 3.5%]
Mean days supplied in discharge opioid prescription[c]	5.4 [4.4, 6.4]	-0.03 [-0.2, 0.08]	-1.1 [-1.8, -0.3]	-0.02 [-0.1, 0.1]
% patients with at least one refill[c]	33.1% [22.5%, 43.7%]	-1.0% [-2.3%, 0.3%]	6.1% [-2.3%, 14.4%]	0.8% [-0.7%, 2.3%]

Non-opioid-naïve patients were those with at least dispensed opioid prescription within the prior 120 days.

[a]Defined as an opioid prescription prescribed at discharge from surgery

[b]Defined as an opioid prescription dispensed within 3 days of discharge from surgery (or within 3 days of discharge from hospitalization after surgery, as applicable)

[c]These 5 outcomes were only calculated for patients with both a discharge and dispensed opioid prescription

© 2023 Chua KP et al. *JAMA Health Forum.*

eTable 3. Characteristics of patients included in the sample and patients excluded from the sample owing to lack of data on patient-reported outcomes

Characteristics	Patients included in the sample	Patients excluded because they lacked complete data on the 3 patient-reported outcomes	*P* value
	**N=6,045**	**N=5,284**	
Age, mean (SD)	48.7 (12.56)	45.5 (12.61)	<0.001
Sex			
Male	2,450 (40.5%)	1,962 (37.1%)	<0.001
Female	3,595 (59.5%)	3,322 (62.9%)	
Race/ethnicity[a]			
Black, non-Hispanic	294 (4.9%)	350 (6.6%)	<0.001
Hispanic	131 (2.2%)	140 (2.6%)	
Other	53 (0.9%)	90 (1.7%)	
White, non-Hispanic	5,182 (85.7%)	4,438 (84.0%)	
Unknown	385 (6.4%)	266 (5.0%)	
Tobacco use	942 (15.6%)	955 (18.1%)	<0.001
Cancer	267 (4.4%)	282 (5.3%)	0.023
BMI			
<18.5	42 (0.7%)	53 (1.0%)	0.504
18.5 to 24.9	1,041 (17.2%)	919 (17.4%)	
25 to 29.9	1,823 (30.2%)	1,580 (29.9%)	
>=30	3,132 (51.8%)	2,726 (51.6%)	
Unknown	7 (0.1%)	6 (0.1%)	
American Society of Anesthesiologists class			
1	789 (13.1%)	677 (12.8%)	0.640
2	3,932 (65.0%)	3,397 (64.3%)	
3	1,281 (21.2%)	1,178 (22.3%)	
4 or 5	38 (0.6%)	29 (0.5%)	
Unknown	5 (0.1%)	3 (0.1%)	
Surgical priority			
Elective	4,646 (76.9%)	4,028 (76.2%)	0.432
Emergent/Urgent	1,399 (23.1%)	1,256 (23.8%)	
Inpatient	2,872 (47.5%)	2,746 (52.0%)	<0.001
Procedure type			
Laparoscopic appendectomy	676 (11.2%)	650 (12.3%)	<0.001
Laparoscopic cholecystectomy	1,663 (27.5%)	1,315 (24.9%)	
Laparoscopic colectomy	195 (3.2%)	187 (3.5%)	
Open colectomy	89 (1.5%)	57 (1.1%)	

© 2023 Chua KP et al. *JAMA Health Forum.*

Minor hernia	1,624 (26.9%)	1,072 (20.3%)	
Major hernia	222 (3.7%)	201 (3.8%)	
Abdominal hysterectomy	232 (3.8%)	215 (4.1%)	
Vaginal hysterectomy	319 (5.3%)	342 (6.5%)	
Laparoscopic hysterectomy	637 (10.5%)	748 (14.2%)	
Other	388 (6.4%)	497 (9.4%)	
Post-operative complications	108 (1.8%)	80 (1.5%)	0.257
Opioid-naïve	5,111 (84.5%)	4,311 (81.6%)	<0.001

[a]Race/ethnicity data are derived from the electronic health record and is typically based on patient self-report. The "other" category includes patients who are American Indian or Alaska Native, Native Hawaiian or Pacific Islander, or Asian.

© 2023 Chua KP et al. *JAMA Health Forum.*
