# Supplementary material for: Changes in Surgical Opioid Prescribing and Patient-Reported Outcomes After Implementation of an Insurer Opioid Prescribing Limit
Source: JAMA Health Forum. 2023 Oct 13;4(10):e233541. doi: 10.1001/jamahealthforum.2023.3541 (PMC10576220; doi:10.1001/jamahealthforum.2023.3541)
Supplement: Supplement 2. — Data Sharing Statement [file jamahealthforum-e233541-s002.pdf]

Data Sharing Statement

Chua. Changes in Surgical Opioid Prescribing and Patient-Reported Outcomes After Implementation of an Insurer Opioid Prescribing Limit. *JAMA Health Forum*. Published October 13, 2023. doi:10.1001/jamahealthforum.2023.3541

Data
Data available: No

Additional Information
Explanation for why data not available: Data from the surgical registry and the prescription drug monitoring program database cannot be released owing to restrictions in the data use agreement.
